# Differential senolytic inhibition of normal versus Aβ-associated cholinesterases: implications in aging and Alzheimer’s disease

**DOI:** 10.18632/aging.206227

**Published:** 2025-03-29

**Authors:** Sultan Darvesh, Meghan K. Cash, Katrina Forrestall, Hillary Maillet, Dane Sands

**Affiliations:** 1 Department of Medical Neuroscience, Dalhousie University, Halifax, Nova Scotia B3H 4R2, Canada; 2 Department of Medicine (Geriatric Medicine and Neurology), Dalhousie University, Halifax, Nova Scotia B3H 2E1 Canada

**Keywords:** cellular senescence, β-amyloid, acetylcholinesterase, butyrylcholinesterase, cholinesterase inhibitors

## Abstract

Cellular senescence is a hallmark of aging and the age-related condition, Alzheimer’s disease (AD). How senescence contributes to cholinergic and neuropathologic changes in AD remains uncertain. Furthermore, little is known about the relationship between senescence and cholinesterases (ChEs). Acetylcholinesterase (AChE) and butyrylcholinesterase (BChE) are important in neurotransmission, cell cycle regulation, and AD amyloid-β (Aβ) pathology. Senolytic agents have shown therapeutic promise in AD models. Therefore, we evaluated *in vitro* and *in silico* activity of senolytics, dasatinib (1), nintedanib (2), fisetin (3), quercetin (4), GW2580 (5), and nootropic, meclofenoxate hydrochloride (6), toward AChE and BChE. As ChEs associated with AD pathology have altered biochemical properties, we also evaluated agents 1-6 in AD brain tissues. Enzyme kinetics showed agents 1, 3, 4, and 6 inhibited both ChEs, while 2 and 5 inhibited only AChE. Histochemistry showed inhibition of Aβ plaque-associated ChEs (1 and 2: both ChEs; 5: BChE; 6: AChE), but not normal neural-associated ChEs. Modeling studies showed 1-6 interacted with the same five binding locations of both ChEs, some of which may be allosteric sites. These agents may exert their beneficial effects, in part, by inhibiting ChEs associated with AD pathology and provide new avenues for development of next-generation inhibitors targeting pathology-associated ChEs.

## INTRODUCTION

The concept of cellular senescence was first used to describe diploid cells that ceased to proliferate [1]. Under normal conditions, cellular senescence has been suggested to be involved in maintaining tissue homeostasis during embryonic development, wound healing and repair, and suppression of tumor proliferation [2]. However, chronic accumulation of these cells can lead to deleterious effects. Cellular senescence is described as a hallmark of aging and age-related conditions such as Alzheimer’s disease (AD) [2, 3].

Cellular senescence is identified by permanent cell cycle arrest and resistance to apoptosis in proliferative cells such as astrocytes, microglia, oligodendrocytes, and endothelial cells [2]. It is independently regulated by the tumor suppressor p53/p21 and p16/pRB pathways [3] that result in preventing G_1_ to S phase transition in the cell cycle [4, 5]. Cellular senescence is triggered by activation of harmful genes and sustained endogenous and exogenous stresses, including DNA and oxidative damage, telomere shortening, mitogenic and oncogenic signaling, mitochondrial dysfunction, and chromatin and lysosomal alterations [2].

Senescent cells are characterized by increased accumulation of the lysosomal enzyme senescence-associated-β-galactosidase [6] and lipofuscin [7], as well as the robust secretion of senescence-associated secretory phenotype (SASP) factors [8] including activated inflammatory cytokines (e.g. interleukin 1β (IL-1β), interleukin 6 (IL-6), and tumor necrosis factor α (TNFα), chemokines, metalloproteinases, extracellular matrix components, and growth factors and regulators [2, 9]. Non-proliferative post-mitotic cells, like neurons, can also exhibit an increase in senescence-associated-β-galactosidase and lipofuscin. They also secrete SASP inflammatory factors [10], the hallmarks of senescence.

For more than 30 years, there has been an interest in the association between cognitive decline and cholinergic neurotransmission in aging, particularly in AD [11–13]. Regarding brain senescence, many facets of the cholinergic system, such as the neurotransmitter acetylcholine, synthesizing enzyme choline acetyltransferase (ChAT) and nicotinic and muscarinic acetylcholine receptors (nAChR and mAChR, respectively), have received great attention [14–18]. However, there have been few studies exploring the relationship between senescence and cholinesterases (ChEs) [18–23].

Acetylcholinesterase (AChE) and butyrylcholinesterase (BChE) are related serine hydrolases that co-regulate the cholinergic system through hydrolysis of acetylcholine [24, 25]. In the normal brain, BChE is primarily expressed in white matter, glia and distinct subcortical populations of neurons important for cognition and behavior while AChE is found in neurons and neuropil throughout the brain, with very little in white matter [24, 26, 27]. Although both ChEs share structural similarities [28] and functional homology [29], both AChE and BChE have distinct functions in health and disease beyond neurotransmission [25, 30].

In normal aging, there is a gradual decline in cholinergic function resulting in reduced levels of acetylcholine, ChAT, nAChRs, mAChRs, and AChE [31]. On the other hand, BChE activity has been shown to increase with age in the normal brain [32]. These changes have been correlated with age-related cognitive decline [11, 33]. In AD, pronounced cholinergic dysfunction is attributed to widespread loss of cholinergic neurons and significantly reduced acetylcholine levels that contribute to the salient cognitive and behavioural deficits characteristic of the disease [34, 35]. In turn, AChE levels are significantly reduced [36], while BChE levels remain the same [37] or increase [36, 38]. AChE and BChE are associated with the pathological hallmarks of AD, amyloid-β (Aβ) plaques and tau neurofibrillary tangles [39, 40], however, these enzymes are not associated with other dementia-related neuropathologies [41–43]. A role for BChE in the maturation of Aβ plaques, and consequently, AD progression [39] has been suggested due to its distinct association with the “malignant” fibrillar plaques of AD brains but not the “benign” non-fibrillar plaques typically found in cognitively normal, aged brains [40, 41]. Plaque-bound ChEs appear to undergo a conformational change that alters their biochemical properties and differentiates these enzymes from those associated with normal neural elements [37, 44–46]. These biochemical changes lead to altered binding affinities [44, 47] and subsequent inhibitor sensitivities between ChEs associated with AD pathology and with normal neural elements [37, 44, 45]. Such alterations have significant implications in the design and development of potential next-generation therapeutic approaches targeting AChE and BChE for treatment of AD.

In addition to termination of cholinergic signaling, BChE is also involved in hydrolysis of the growth hormone secretagogue ghrelin, drug hydrolysis, lipid metabolism, detoxification of xenobiotics, and protein interaction and modification [25, 48, 49]. Moreover, both ChEs are also involved in cell proliferation and neural development [50, 51]. The non-cholinergic functions of AChE and BChE have been attributed to sharing similar sequence homologies of cell adhesion and ChE-like cell adhesion molecules [52, 53]. However, under pathological conditions, cell proliferation and apoptotic functions of ChEs appear altered. For instance, high levels of BChE activity and *BCHE* expression have been implicated in the rapid undifferentiated cellular proliferation of several brain tumor types [54, 55]. However, this rate of proliferation was reduced through the abolition of *BCHE* and BChE enzyme [55]. The *BCHE* gene has been shown to be upregulated in cell immortality in tumor development, whereby unchecked cell proliferation and evasion of cell death prevail, however, BChE suppression was shown to increase the rate of apoptotic processes [56].

Many approved and experimental cancer therapies are pro-apoptotic agents that exert their effects by targeting several intrinsic and extrinsic signaling pathways, including tumor suppressor pathways [57]. Furthermore, a number of these anti-cancer agents have been repurposed as senolytic agents as they also exhibit suppression of cellular senescence [58]. However, only a few senolytics have been identified as potential ChE inhibitors (ChEIs) [59, 60]. ChEIs that target AChE, BChE, or both [61], are a class of drugs that have been widely used for the treatment of several conditions, most notably the cognitive and behavioral symptoms associated with AD [62]. Several ChEIs, such as the AChE-selective donepezil and galantamine and the BChE-selective rivastigmine have shown to modulate apoptotic pathways and exert potent anti-inflammatory effects, in addition to their primary mode of action of increasing acetylcholine levels [63–65].

Cellular senescence is a significant contributor to low levels of chronic inflammation in normal aging, with notably increased levels of inflammatory cytokines in AD [2]. There is an established relationship between cholinergic function and immunomodulation [66–69]. Immune cells are equipped with the necessary cholinergic components, including ChAT, nAChRs and mAChRs, as well as AChE and BChE, required for modulation of cell activity through the cholinergic anti-inflammatory pathway (CAIP). ChEIs stimulate CAIP [70, 71], leading to decreases in macrophage and microglial secretion of pro-inflammatory cytokines such TNFα, IL-1β, and IL-6 [66, 67, 72].

**Figure 1:**
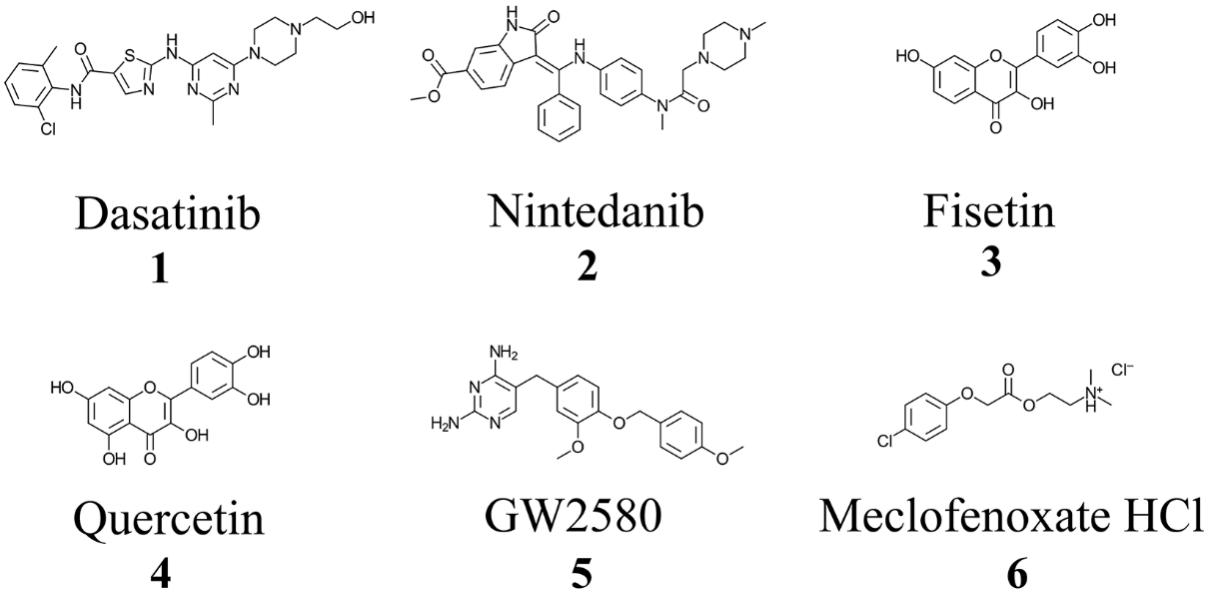
**Chemical structures of approved and investigational senolytic (1-5) and nootropic (6) agents.** Structures were drawn using ChemDoodle: 2D Chemical Drawing, Publishing and Informatics (Version 11.10, iChemLabs, LLC, Somerset, NJ, USA).

Senolytic agents such as dasatinib, nintedanib, fisetin, and quercetin and the nootropic, anti-aging agent meclofenoxate hydrochloride (also referred to as centrophenoxine) (Figure 1) have undergone or are currently undergoing human clinical trials in patients with age-related diseases [73–75]. They, along with the senolytic GW2580, have also had promising results in ameliorating inflammation, decreasing pathological load, and improving cognition in preclinical models of aging and AD [76–84]. However, the mechanism(s) by which senescent cells and their pro-inflammatory SASP factors contribute to aging and neurodegeneration, including neuropathology and dysfunction of the cholinergic system and CAIP is unknown. It has been suggested that increasing the bioavailability of acetylcholine for CAIP activation, via ChE inhibition, could be an attractive avenue for decreasing neuroinflammation and modulating apoptotic activity in aging and AD [72, 85].

Because of the importance of ChEs in cell communication, the cell cycle, CAIP and AD neuropathology, we evaluated the enzyme kinetic properties of several senolytic and nootropic agents towards AChE and BChE to determine if they were inhibitors of these enzymes. In addition, since biochemical properties of ChEs associated with AD pathology are different from those associated with normal neural elements, using histochemical methods, we also evaluated whether these compounds inhibited ChEs that are associated with Aβ plaques in AD. We show that the selected senolytics and nootropic inhibit ChEs associated with plaques but not the enzymes associated with normal neural elements. Together, these findings suggest that these senolytic agents may exert their beneficial effects, in part, by inhibiting ChE-associated with Aβ pathology and providing new avenues for the development of the next generation of ChE inhibitors targeting AD pathology-associated ChEs.

## RESULTS

### Enzyme kinetic studies

**Figure 2:**
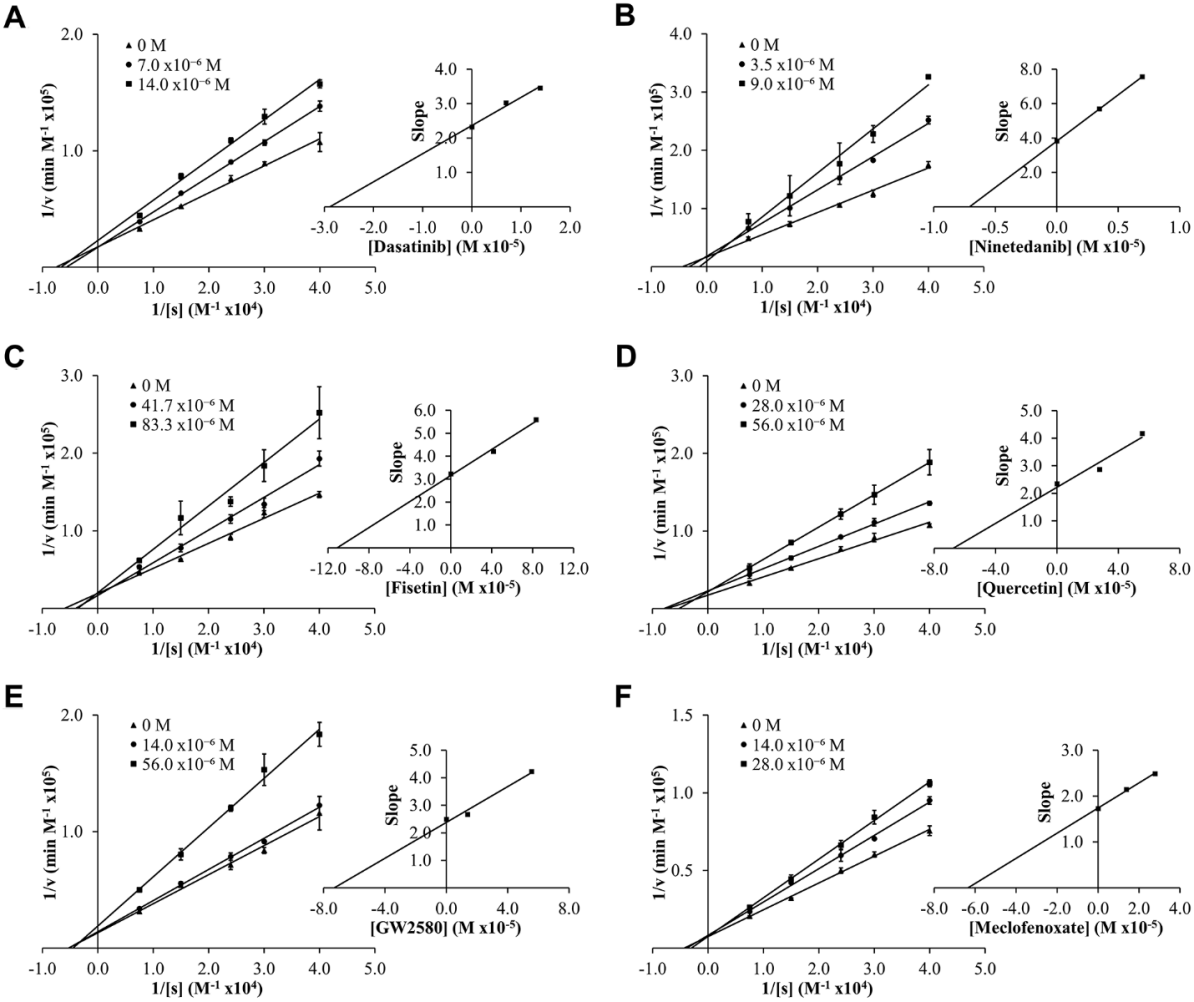
Lineweaver-Burk (LB) plots showing inhibition of acetylcholinesterase (AChE) upon treatment with varying concentrations of senolytic and nootropic agents 1-6 (**A**–**F**, respectively). Each compound was assessed at a range of concentrations (0-83 μM), with 0 M (▲), middle concentration (●), and highest concentration (■). Slopes from LB plot trendlines were plotted against compound concentrations to generate *K*_i_ values as the x-intercept (insert graphs). Enzyme kinetic experiments were performed in triplicate and kinetic parameter values were averaged.

**Figure 3:**
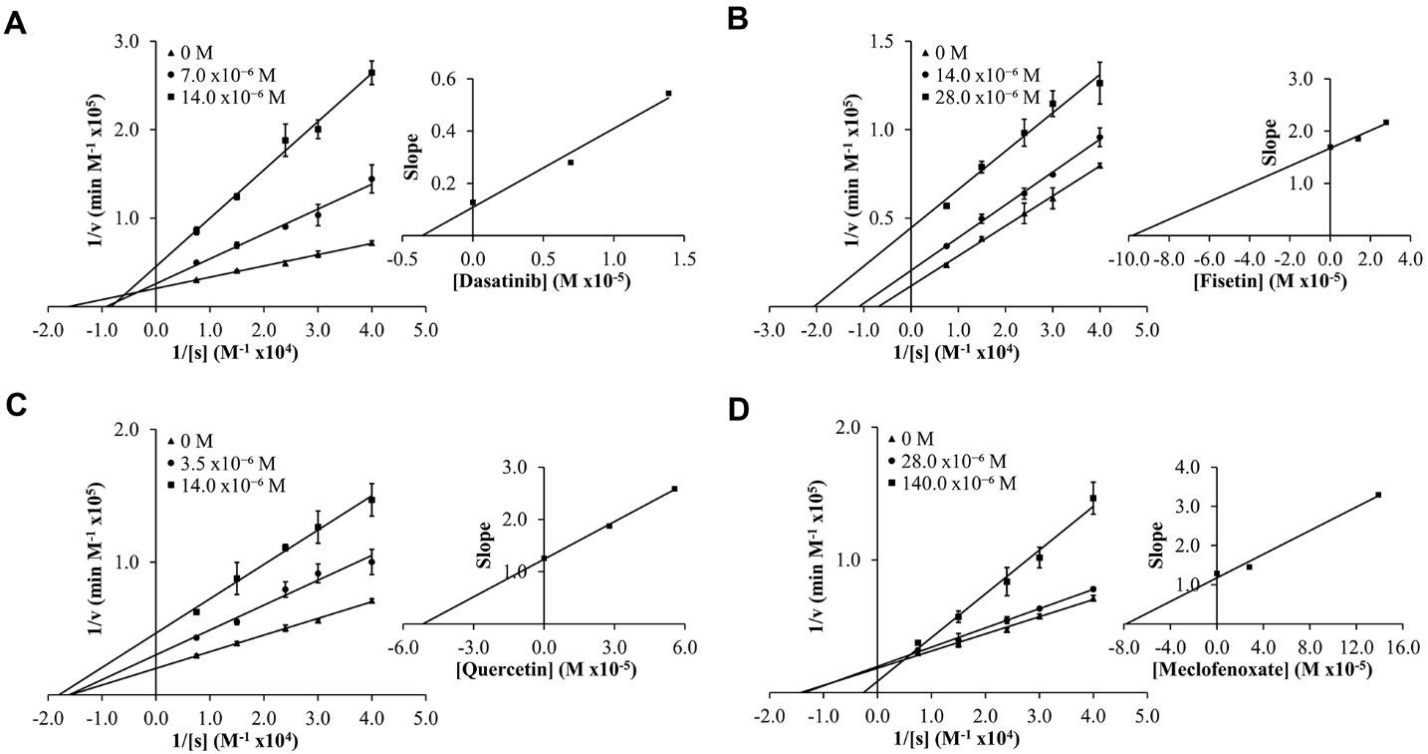
Lineweaver-Burk (LB) plots showing inhibition of butyrylcholinesterase (BChE) upon treatment with varying concentrations of senolytic and nootropic agents 1, 3, 4 and 6 (**A**–**D**, respectively). Compounds 2 and 5 showed too little inhibition to complete LB kinetics. Each compound was assessed at a range of concentrations (0-140 μM), with 0 M (▲), middle concentration (●), and highest concentration (■). Slopes from LB plot trendlines were plotted against compound concentrations to generate *K*_i_ values as the x-intercept (insert graphs). Enzyme kinetic experiments were performed in triplicate and kinetic parameter values were averaged.

To determine experimental inhibition constants (*K*_i_ values) and mode of inhibition for senolytic and nootropic compounds 1-6 with ChEs, Lineweaver-Burk plots were generated (Figures 2, 3), as described previously [86]. Calculated experimental *K*_i_ values (μM) are shown in Table 1. Where ChE inhibition constants for compounds were identified previously in the literature [60, 87], *K*_i_ values observed herein were also similar (Table 1). All compounds showed some degree of ChE inhibition, except for 2 and 5 which did not inhibit BChE. Nintedanib (2) showed activity that was too low to conduct a full inhibition study.

**Table 1:** Experimental inhibition constants (*K*_i_ values) and mode of inhibition for compounds 1-6 with acetylcholinesterase (AChE) and butyrylcholinesterase (BChE).

**Compound**	**Experimental AChE *K*_i_ (μM) (Mode of inhibition)Literature AChE *K*_i_ values**	**Experimental BChE *K*_i_ (μM) (Mode of inhibition) Literature BChE *K*_i_ values**
Dasatinib (**1**)	28.22	3.36
	(Mixed Non-competitive)	(Mixed Non-competitive)
Nintedanib (**2**)	7.10	No Inhibition
	(Mixed Non-competitive)	
Fisetin (**3**)	109.81	94.35
	(Competitive)	(Uncompetitive)
	100.2^a^	94.2^a^
Quercetin (**4**)	62.19	51.49
	(Mixed Non-competitive)	(Mixed Non-competitive)
	38.3^a^, 76.2^b^	68.0^a^, 46.8^b^
GW2580 (**5**)	70.97	No Inhibition
	(Mixed Non-competitive)	
Meclofenoxate Hydrochloride (**6**)	63.20	76.30
	(Mixed Non-competitive)	(Mixed Non-competitive)

^a^*K*_i_ values taken from Katalinić et al. [60].

^b^*K*_i_ values taken from Orhan et al. [87].

Modes of inhibition were determined from Lineweaver-Burk plots, with most compounds showing mixed non-competitive inhibition, often indicative of allosteric binding – double reciprocal plot trendlines crossing between x- and y-axes (Figures 2, 3). Fisetin (3) was the exception, showing robust competitive inhibition for AChE – trendlines crossing at the y-axis (Figure 2C), and clear uncompetitive inhibition with BChE – trendlines are parallel (Figure 3B). These results show that 3 outcompetes acetylthiocholine iodide (ATChI) for the active site of AChE; while 3 only targets the enzyme-butyrylthiocholine iodide (BTChI) complex at which point it could bind to the active or allosteric sites to render inhibition of BChE. All other senolytic and nootropic agents are likely binding to only allosteric sites or a combination of active and allosteric sites, to produce the observed mixed non-competitive inhibitory responses.

### Histochemical studies

Of the AD brains selected for this study (Table 2), three cases were previously described to have robust Aβ-, AChE-, and BChE-associated plaque loads in the cerebral cortex [42, 46, 88]. The fourth case, BB11-010, also showed severe AD and ChE-associated pathology, consistent with the other selected cases.

**Table 2:** Demographic information for Alzheimer’s disease (AD) brain tissues used.

**Case #**	**Neuropathological diagnosis**	**Sex**	**Age (y)**	**Brain weight (g)**	**Braak stage**	**CERAD^a^ plaque score**
BB11-010	AD	M	93	1254	IV	Frequent
BB11-026	AD	M	98	1066	V	Frequent
BB11-039	AD	F	92	1009	IV	Frequent
BB12-035	AD	F	84	1120	VI	Frequent

^a^CERAD, Consortium to Establish a Registry for Alzheimer’s Disease

Histochemical staining was done at pH 6.8 to allow for the visualization of ChE activity associated with plaques, while pH 8.0 was used to visualize ChE activity associated with normal neural elements, as shown previously [37, 88]. The staining quality of ChEs in tissues selected for this study were examined using the standard Karnovsky-Roots (KR) substrate staining method and found to be appropriate prior to treatment with compounds 1-6.

**Figure 4:**
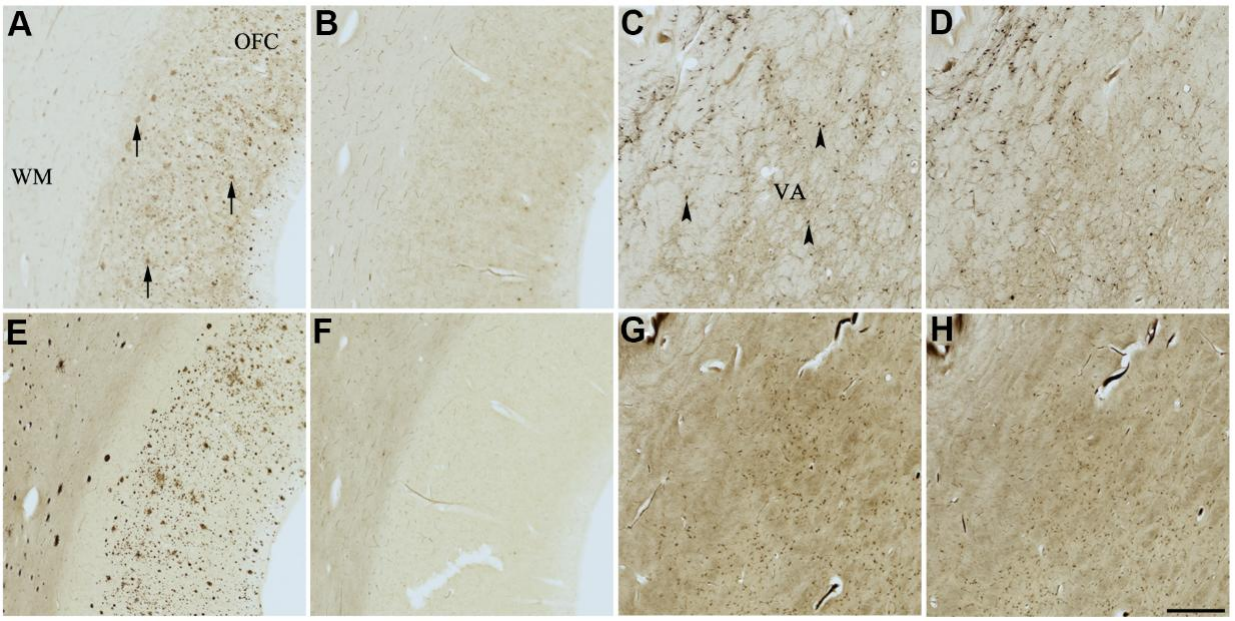
**Effect of dasatinib (1) on histochemical staining of acetylcholinesterase (AChE) and butyrylcholinesterase (BChE).** Representative photomicrographs of histochemical staining of AChE (**A**–**D**) and BChE (**E**–**H**). Staining at pH 6.8 demonstrates AChE- (**A**) and BChE (**E**)-associated plaques in the Alzheimer’s disease (AD) orbitofrontal cortex (arrows). Staining at pH 8.0 demonstrates AChE (**C**) and BChE (**G**) associated with normal neural structures in the AD thalamus (arrowheads showing neurons). Dasatinib (1) inhibits AChE (**B**) and BChE (**F**) associated with AD plaques but not AChE (**D**) and BChE (**H**) associated with normal neural elements. Note, for ease of reference, identical images of the positive control staining of AChE and BChE at pH 6.8 and 8.0 (**A**, **C**, **E**, **G**) were used herein and in Figures 5-9 (**A**, **C**, **E**, **G**) to help compare directly the effects of each senolytic or nootropic agent on the standard Karnovsky-Roots (KR) histochemical staining method. Abbreviations: OFC, orbitofrontal cortex; VA, ventroanterior thalamic nucleus; WM, white matter. Scale bar = 500 μm.

**Figure 5:**
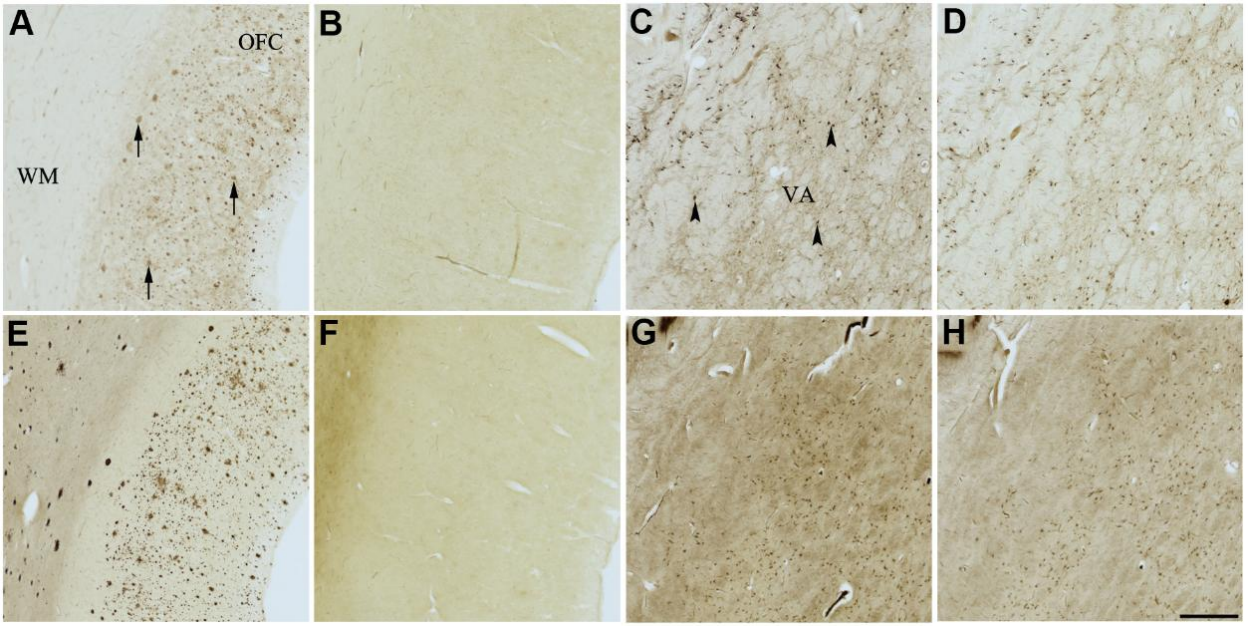
**Effect of nintedanib (2) on histochemical staining of acetylcholinesterase (AChE) and butyrylcholinesterase (BChE).** Representative photomicrographs of histochemical staining of AChE (**A**–**D**) and BChE (**E**–**H**). Staining at pH 6.8 demonstrates AChE- (**A**) and BChE (**E**)-associated plaques in the Alzheimer’s disease (AD) orbitofrontal cortex (arrows). Staining at pH 8.0 demonstrates AChE (**C**) and BChE (**G**) associated with normal neural structures in the AD thalamus (arrowheads showing neurons). Nintedanib (2) inhibits AChE (**B**) and BChE (**F**) associated with AD plaques but not AChE (**D**) and BChE (**H**) associated with normal neural elements. Note, for ease of reference, identical images of the positive control staining of AChE and BChE at pH 6.8 and 8.0 (**A**, **C**, **E**, **G**) were used herein and in Figures 4, 6–9 (**A**, **C**, **E**, **G**) to help compare directly the effects of each senolytic or nootropic agent on the standard Karnovsky-Roots (KR) histochemical staining method. Abbreviations: OFC, orbitofrontal cortex; VA, ventroanterior thalamic nucleus; WM, white matter. Scale bar = 500 μm.

**Figure 6:**
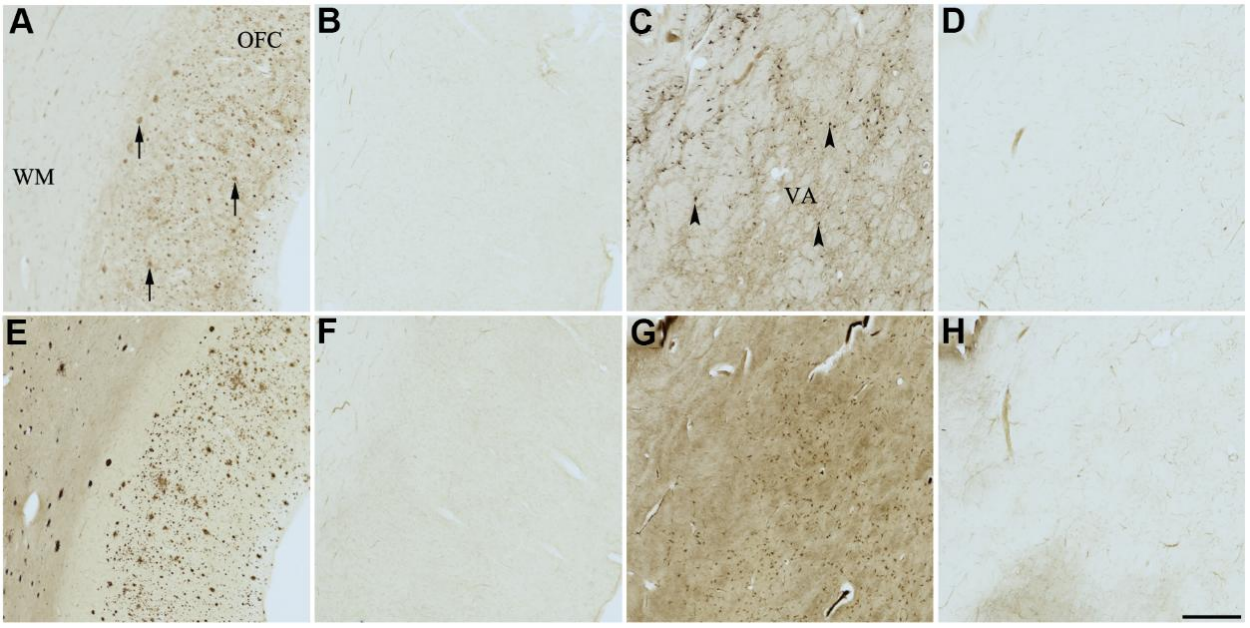
**Effect of fisetin (3) on histochemical staining of acetylcholinesterase (AChE) and butyrylcholinesterase (BChE).** Representative photomicrographs of histochemical staining of AChE (**A**–**D**) and BChE (**E**–**H**). Staining at pH 6.8 demonstrates AChE- (**A**) and BChE (**E**)-associated plaques in the Alzheimer’s disease (AD) orbitofrontal cortex (arrows). Staining at pH 8.0 demonstrates AChE (**C**) and BChE (**G**) associated with normal neural structures in the AD thalamus (arrowheads showing neurons). Staining reactions for AChE and BChE at pH 6.8 (**B** and **F**) or pH 8.0 (**D** and **H**) in the presence of fisetin (3) could not be done due to compound 3 precipitating out of solution, most likely due to chelating with metal ions required for the Karnovsky-Roots (KR) staining method. Note, for ease of reference, identical images of the positive control staining of AChE and BChE at pH 6.8 and 8.0 (**A**, **C**, **E**, **G**) were used herein and in Figures 4, 5, 7–9 (**A**, **C**, **E**, **G**) to help compare directly the effects of each senolytic or nootropic agent on the standard KR histochemical staining method. Abbreviations: OFC, orbitofrontal cortex; VA, ventroanterior thalamic nucleus; WM, white matter. Scale bar = 500 μm.

**Figure 7:**
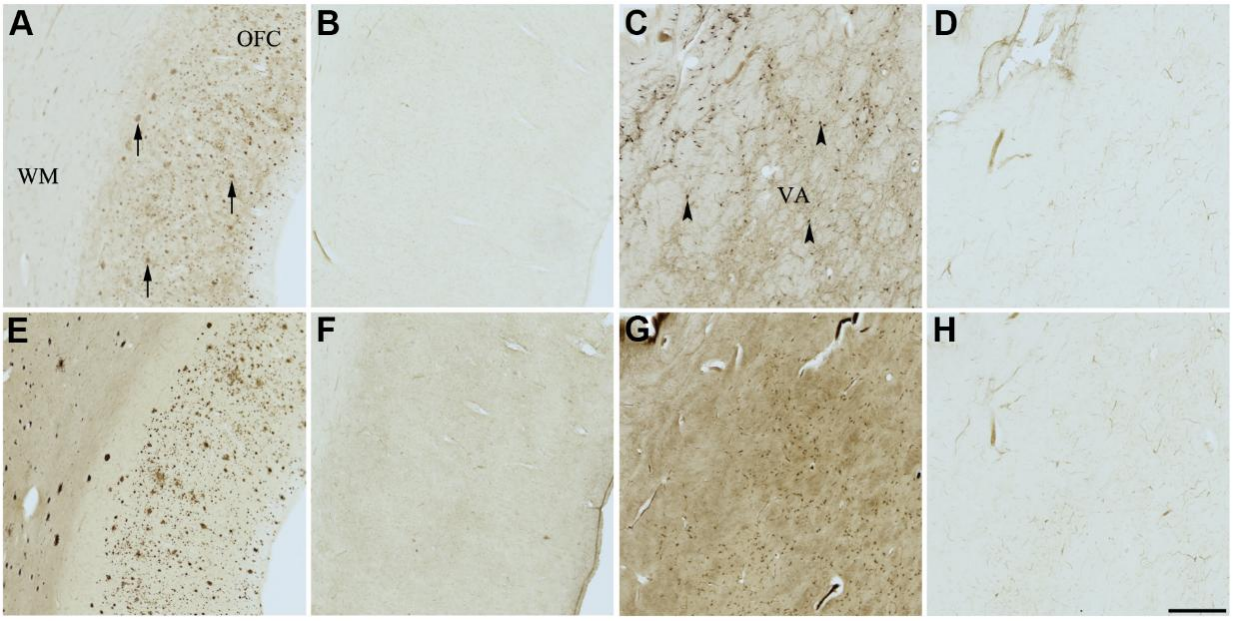
**Effect of quercetin (4) on histochemical staining of acetylcholinesterase (AChE) and butyrylcholinesterase (BChE).** Representative photomicrographs of histochemical staining of AChE (**A**–**D**) and BChE (**E**–**H**). Staining at pH 6.8 demonstrates AChE- (**A**) and BChE (**E**)-associated plaques in the Alzheimer’s disease (AD) orbitofrontal cortex (arrows). Staining at pH 8.0 demonstrates AChE (**C**) and BChE (**G**) associated with normal neural structures in the AD thalamus (arrowheads showing neurons). Staining reactions for AChE and BChE at pH 6.8 (**B** and **F**) or pH 8.0 (**D** and **H**) in the presence of quercetin (4) could not be done due to compound 4 precipitating out of solution, most likely due to chelating with metal ions required for the Karnovsky-Roots (KR) staining method. Note, for ease of reference, identical images of the positive control staining of AChE and BChE at pH 6.8 and 8.0 (**A**, **C**, **E**, **G**) were used herein and in Figures 4–6, 8, 9 (**A**, **C**, **E**, **G**) to help compare directly the effects of each senolytic or nootropic agent on the standard BChE KR histochemical staining method. Abbreviations: OFC, orbitofrontal cortex; VA, ventroanterior thalamic nucleus; WM, white matter. Scale bar = 500 μm.

**Figure 8:**
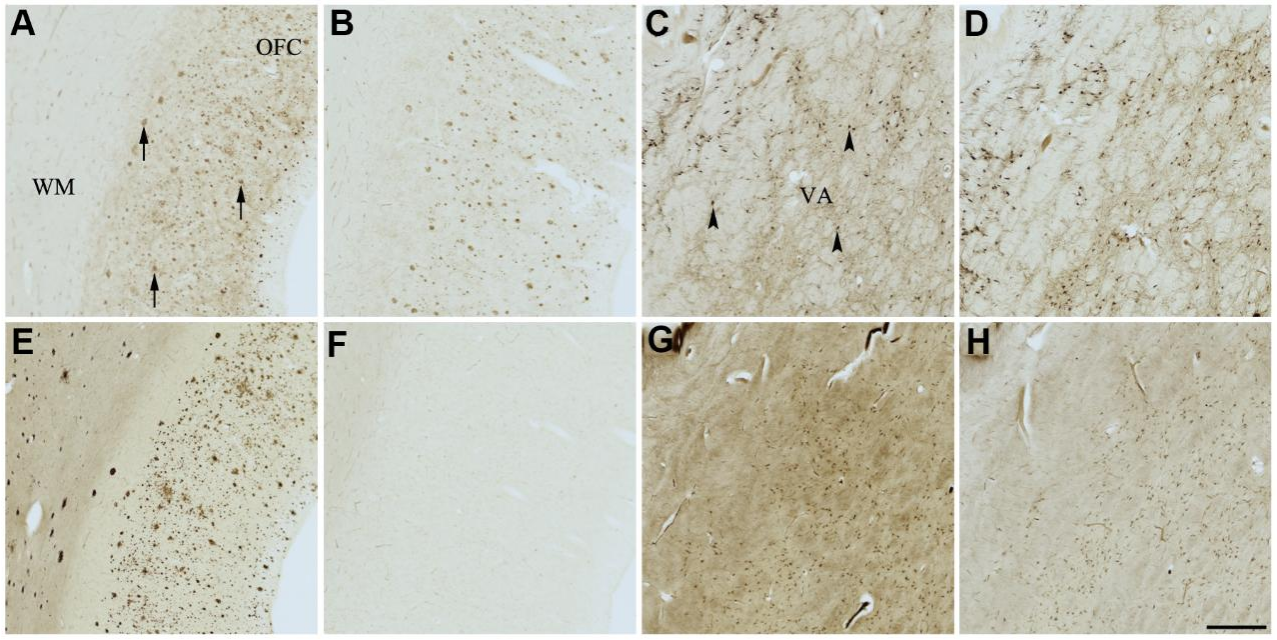
**Effect of GW2580 (5) on histochemical staining of acetylcholinesterase (AChE) and butyrylcholinesterase (BChE).** Representative photomicrographs of histochemical staining of AChE (**A**–**D**) and BChE (**E**–**H**). Staining at pH 6.8 demonstrates AChE- (**A**) and BChE (**E**)-associated plaques in the Alzheimer’s disease (AD) orbitofrontal cortex (arrows). Staining at pH 8.0 demonstrates AChE (**C**) and BChE (**G**) associated with normal neural structures in the AD thalamus (arrowheads showing neurons). GW2580 (5) inhibits BChE (**F**) but not AChE (**B**) associated with AD plaques but not AChE (**D**) and BChE (**H**) associated with normal neural elements. Note, for ease of reference, identical images of the positive control staining of AChE and BChE at pH 6.8 and 8.0 (**A**, **C**, **E**, **G**) were used herein and in Figures 4–7, 9 (**A**, **C**, **E**, **G**) to help compare directly the effects of each senolytic or nootropic agent on the standard Karnovsky-Roots (KR) histochemical staining method. Abbreviations: OFC, orbitofrontal cortex; VA, ventroanterior thalamic nucleus; WM, white matter. Scale bar = 500 μm.

**Figure 9:**
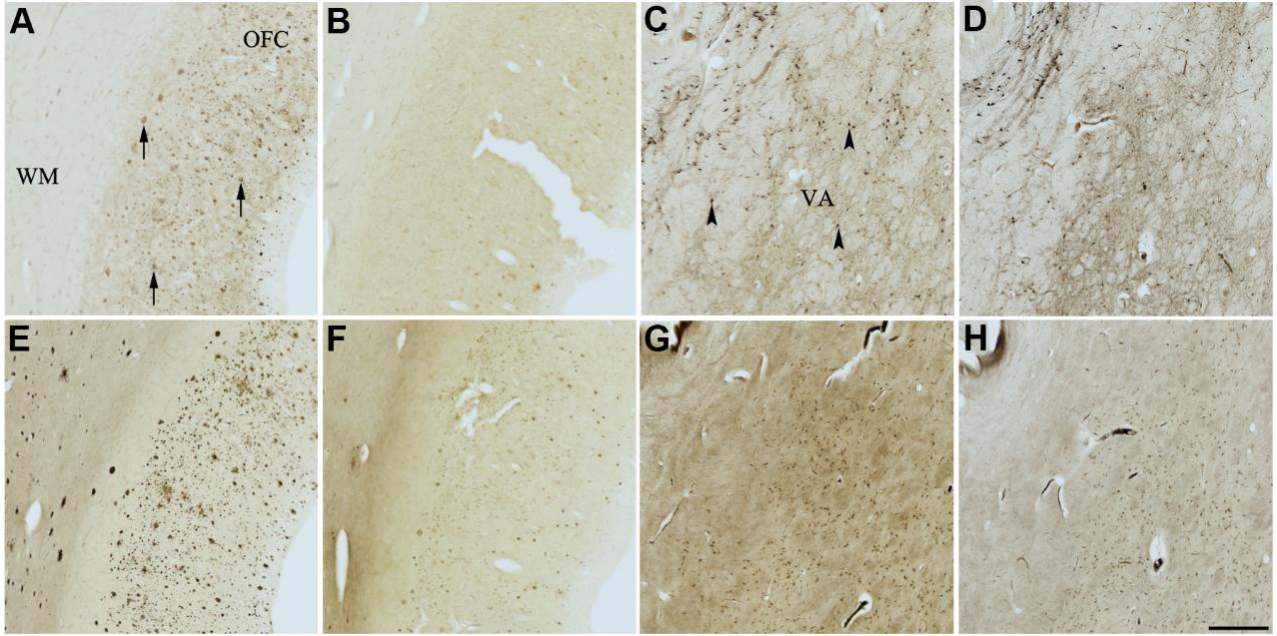
**Effect of meclofenoxate hydrochloride (6) on histochemical staining of acetylcholinesterase (AChE) and butyrylcholinesterase (BChE).** Representative photomicrographs of histochemical staining of AChE (**A**–**D**) and BChE (**E**–**H**). Staining at pH 6.8 demonstrates AChE- (**A**) and BChE (**E**)-associated plaques in the Alzheimer’s disease (AD) orbitofrontal cortex (arrows). Staining at pH 8.0 demonstrates AChE (**C**) and BChE (**G**) associated with normal neural structures in the AD thalamus (arrowheads showing neurons). Meclofenoxate hydrochloride (6) inhibits AChE (**B**) and, to a certain extent, BChE (**F**) associated with AD plaques but not AChE (**D**) and BChE (**H**) associated with normal neural elements. Note, for ease of reference, identical images of the positive control staining of AChE and BChE at pH 6.8 and 8.0 (**A**, **C**, **E**, **G**) were used herein and in Figures 4-8 (**A**, **C**, **E**, **G**) to help compare directly the effects of each senolytic or nootropic agent on the standard Karnovsky-Roots (KR) histochemical staining method. Abbreviations: OFC, orbitofrontal cortex; VA, ventroanterior thalamic nucleus; WM, white matter. Scale bar = 500 μm.

The use of senolytic and nootropic compounds 1-6 in KR histochemical staining resulted in varying degrees of inhibition of AChE and BChE activity associated with normal neural elements (pH 8.0) and plaques (pH 6.8) in human AD brain tissues, ranging from no inhibition (-) to strong inhibition (xxx). Qualitative analysis of each of the four AD cases selected is described as the average rankings for each compound in Table 3. Representative photomicrographs showing AChE and BChE histochemical staining of AD brain tissues with or without treatment with compounds 1-6 are shown in Figures 4–9.

**Table 3:** Qualitative analysis of senolytic and nootropic compound inhibition of Karnovsky-Roots staining for acetylcholinesterase (AChE) and butyrylcholinesterase (BChE) associated with β-amyloid plaques (pH 6.8) and normal neural elements (pH 8.0) in human orbitofrontal cortex and thalamus Alzheimer’s disease brain tissues.

**Senolytic compound**	**Staining results**			
	**AChE pH 6.8**	**BChE pH 6.8**	**AChE pH 8.0**	**BChE pH 8.0**
Dasatinib (**1**)	xx/x^a^	xxx	-	-
Nintedanib (**2**)	xxx	xxx	-	-
Fisetin (**3**)	See below^b^	See below^b^	See below^b^	See below^b^
Quercetin (**4**)	See below^b^	See below^b^	See below^b^	See below^b^
GW2580 (**5**)	-	x/xx	-	-
Meclofenoxate Hydrochloride (**6**)	xx/x	x	-	-

^a^The inhibition in AChE or BChE staining resulting from senolytic and nootropic compound competition was categorized as: – (no inhibition), x (slight inhibition), xx (moderate inhibition), or xxx (strong inhibition).

^b^The presence of these compounds in the reaction mixture led to the chelation and precipitation of metal ions necessary for the Karnovsky-Roots method. Thus, histochemical staining could not be done.

In the presence of compound 1, AChE staining associated with plaques was reduced by varying degrees when compared with control staining at pH 6.8 (Table 3 and Figure 4A, 4B). While two of the AD cases showed complete inhibition of AChE-associated plaques, the remaining cases only showed moderate reduction in staining. Compound 1 showed complete inhibition of BChE activity associated with plaques in all cases, although white matter staining remained when compared to control staining at pH 6.8 (Table 3 and Figure 4E, 4F). There was no inhibition of AChE or BChE staining associated with normal neural elements (pH 8.0) in any cases, relative to controls, when treated with compound 1 (Figure 4C, 4D, 4G, 4H).

Similarly, complete inhibition of both AChE and BChE activity associated with plaques, compared to controls, was observed in the presence of compound 2 (Figure 5A, 5B, 5E, 5F). There was no inhibition of ChE staining associated with normal neural elements (Table 3 and Figure 5C, 5D, 5G, 5H).

The two flavonoids, compounds 3 and 4, produced identical results under KR histochemical conditions (Table 3 and Figures 6, 7). When the aqueous stock solutions of 3 and 4 were added to their KR staining solutions, there were noticeable color and solubility changes from the original stocks. The stock solution of compound 3 was a cloudy, peach color, which turned umber and precipitated upon addition to the KR solution. The stock solution of compound 4 was a cloudy, neon yellow color, which turned brown and likewise precipitated when added to the KR solution. These observations indicated that the lack of ChE staining at pH 6.8 and 8.0 for tissues tested with compounds 3 and 4 (Figures 6, 7) were likely due to both flavonoids forming complexes with metal ions of the KR solution, iron and/or copper [89, 90], necessary for the staining reaction to occur. Thus, producing the false impression of inhibitory activity.

Compound 5 did not inhibit staining of AChE associated with plaques; however, it did inhibit staining of BChE associated with plaques when compared to control staining (Table 3 and Figure 8A, 8B, 8E, 8F). Furthermore, compound 5 did not inhibit either ChE associated with normal neural elements compared to controls (Table 3 and Figure 8C, 8D, 8G, 8H).

Lastly, compound 6 showed moderate to strong inhibition of AChE-associated plaque staining and slight inhibition of BChE-associated plaque staining when compared to control staining for AChE and BChE, respectively (Figure 9A, 9B, 9E, 9F). Although reduced, the appearance of AChE inhibition associated with plaques varied when comparing all AD cases. Cases with a relatively higher plaque burden appeared to have less reduction in ChE staining than cases with a relatively lower plaque burden. When comparing appropriate control sections to those stained in the presence of compound 6, the degree of inhibition remained the same in amongst all cases (not shown). Compound 6 did not inhibit AChE or BChE staining associated with normal neural elements (Table 3 and Figure 9C, 9D, 9G, 9H).

### Molecular docking studies

Cholinesterase inhibitors are known to bind to varying sites on ChEs to yield effects on catalytic activity. *In silico* molecular docking studies provide a means of assessing potential enzyme binding sites and amino acid residue interactions that could contribute to the observed activity of compounds. As such, understanding the structural characteristics and features of AChE and BChE is essential to appropriately investigate the potential mechanisms of action for active compounds. Structural features of ChEs used to analyze molecular docking studies were based on the published x-ray crystal structures of these enzymes [91, 92].

**Figure 10:**
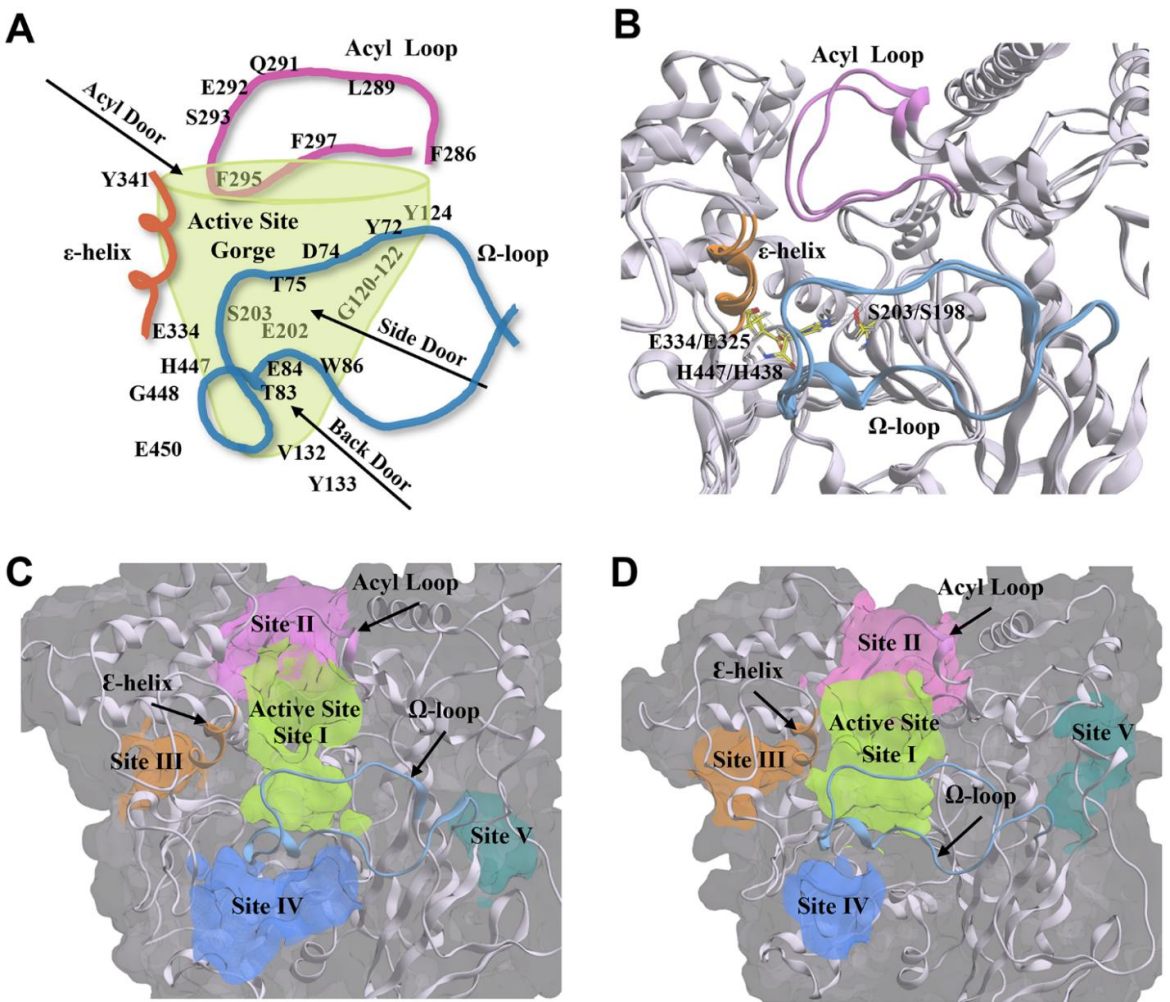
**Structural components and amino acid residues that are essential for the catalytic activity of acetylcholinesterase (AChE) and butyrylcholinesterse (BChE**). (**A**) 2D depiction of the entry/exit routes (“doors”) to the active site gorge of AChE. Amino acids are labelled and arranged according to their structural placement around the gorge, with those located on the opposite side to the viewing plane shown through the transparent green gorge wall. (**B**) AChE (PDB: 4M0E, 2.00 Å) [91] and BChE (BChE; PDB: 4TPK, 2.70 Å) [92] enzyme ribbon structures are overlayed to show structural conservation between the two enzymes. The catalytic triad residues are shown in yellow. (**C**, **D**) The five main compound binding site pockets (I-V) of AChE (**C**) and BChE (**D**) are identified as the enzyme active site gorge (lime green, I), pocket behind ChE acyl loop (pink, II), pocket behind ChE catalytic glutamate (orange, III), pocket behind ChE active site tryptophan (Back Door, blue, IV), and other binding pocket (teal, V). Structural features of AChE and BChE including the acyl loop (pink), Ω-loop (blue), and ε-helix (orange) are shown throughout each panel. Figures were generated using Microsoft PowerPoint for Microsoft 365 MSO (version 2409 build 18025.20104; Microsoft Corporation, Redmond, WA, USA) and Molecular Operating Environment 2022.02 (Chemical Computing Group ULC, Montreal, Quebec, Canada).

AChE and BChE possess many of the same structural components and similar amino acid residues that are essential for catalytic activity. Though the volume of the active site gorge and the diameter of the gorge opening are larger in BChE than in AChE (~500 Å^3^ vs. ~300 Å^3^, ~16 Å vs. ~11 Å) [93], they both have a similar depth of ~20 Å and contain the same five main active site regions with key residues and structural features (acyl loop, Ω-loop, ε-helix) shown in Figure 10. These five regions include: 1) the catalytic active site (CAS) at the base of the gorge, comprised of the catalytic triad, serine (S203 for AChE, S198 for BChE), histidine (H447, H438), and glutamate (E334, E325); 2) the acyl binding pocket (ABP) at the mouth of the gorge; 3) the peripheral anionic site (PAS) at the mouth of the gorge; 4) the π-cationic site (PCS) midway down the gorge; and 5) the oxyanion hole (OAH) next to the catalytic serine at the bottom of the gorge (Supplementary Figure 1).

In addition to the main active site gorge, there are several proposed sites that are entry/exit routes for substrates and hydrolysis products that surround and share some residues of the active site gorge, the acyl and Ω-loops, and the ε-helix. These sites are denoted as the acyl, side, and back doors (Figure 10A) [94–97]. The discovery of the back door was originally hypothesized as an alternate exit route for choline from the active site of AChE following the hydrolysis of acetylcholine, and offered a possible explanation for its high turnover rate despite the restricted opening of the active site gorge [97]. The side and acyl doors were later proposed as additional entry/exit points to the gorge (Figure 10A) [94, 96, 98]. While these “doors” have not been described in detail in the literature for BChE, their key residues and structural components are likewise present in the BChE structure (Figure 10B), with many of the residues conserved between the two enzymes.

Utilizing a blind docking approach [99], with five docking poses generated in each docking experiment and repeated in triplicate, compounds 1-6 localized to the same five main ChE sites (Figure 10C, 10D) identified using the Molecular Operating Environment (MOE) *Site Finder* feature. These five sites are denoted as follows: (I) enzyme active site gorge, (II) pocket behind ChE acyl loop, (III) pocket behind ChE catalytic glutamate and Ɛ-helix, (IV) pocket behind key ChE active site tryptophan (“Back Door”), and (V) other site located on the opposite side of the enzyme, away from the active site gorge. While blind docking experiments with compounds 1-6 did not show all five of these sites in each experiment, compound blind docking poses were distributed between these five ChE enzyme locations (Supplementary Table 1). These five binding pockets are displayed for each ChE crystal structure in Figure 10C, 10D.

Senolytics 1 and 2 did not find the active site of either ChE enzyme in blind docking experiments (Supplementary Table 1). Compounds 3-6 localized to the active site of AChE, while compounds 3, 4, and 6 showed affinities for the active site of BChE (Supplementary Table 1). Compound 5 did not find the active site of BChE. Though compounds 3, 4, and 6 found the active site of both enzymes, only quercetin (4) with BChE and fisetin (3) with AChE showed predominant affinity for the active site gorge in blind docking poses (Supplementary Table 1). Compounds that did not find the active site (1, 2 for AChE; 1, 2, 5 for BChE) – or those that did not localize to the active site in the majority of blind docking poses (4, 5 for AChE; 3 for BChE) – showed affinity most frequently to one major site on each enzyme, site II for AChE and site III for BChE (Supplementary Table 1). Meclofenoxate hydrochloride (6) with both ChEs, and nintedanib (2) with AChE showed equal affinity (6/15 poses) for two sites. For compound 2, these sites were II and III of AChE (Supplementary Table 1), while for 6 these were sites I and III with BChE, and I and IV with AChE.

While it remains uncertain if the frequency of localization of blind docking poses to specific enzyme sites is indicative of true compound preference for that site, or simply a result of varying factors related to the blind modeling process [99–101], it is generally accepted that the failure of a compound to find the active site of an enzyme during blind docking experiments is predictive of the compound not interacting with that active site experimentally [99, 102–104]. Localization to sites beyond the active site gorge of an enzyme may indicate compound affinity for allosteric binding sites; thus, all compounds were assessed in site-directed docking experiments at all identified ChE sites. This resulted in some compounds having multiple calculated inhibition constants (*K*_i_ values) for different binding sites, which are summarized in the Supplementary Materials (Supplementary Table 2).

Predicted *K*_i_ values were generally within the same order of magnitude as experimentally determined *K*_i_ values but were highly variable depending on the enzyme site used for site-directed docking. For example, dasatinib (1) docked with BChE at site II had a sub-micromolar predicted *K*_i_, while the dock directed to site III showed a predicted *K*_i_ that was almost identical to the experimental *K*_i_ value (Supplementary Table 2). Similarly, fisetin (3) and quercetin (4) found the active sites of both ChEs in blind docks but predicted *K*_i_ values at these sites were much lower than experimental values. However, the predicted *K*_i_ values at alternate sites for compounds 3 and 4 were closer to their experimental values (Table 1 and Supplementary Table 2). For AChE, site II consistently showed *K*_i_ values closest to those determined experimentally (Table 1 and Supplementary Table 2). For BChE, there was more variability in alternate sites, but site III produced more accurate *K*_i_ values for several compounds (Table 1 and Supplementary Table 2).

Key amino acid interactions between compounds 1-6 and residues of each ChE were analyzed for the top site-directed docking pose at each identified site (I-V; Supplementary Table 3). Their interaction type, bond lengths, and residues’ location on the enzyme were identified, with most being hydrogen bonding interactions. Overall, similar amino acid residue interactions were observed for compounds docked to the same site (I-V). Likewise, many of these residues were components of the acyl and Ω-loops, the Ɛ-helix, or were residues of the five main regions of the ChE active site gorge (CAS, ABP, PAS, PCS, and OAH).

## DISCUSSION

In normal aging, as well as in age-related diseases such as AD, there is abnormal accumulation of senescent cells in the brain [2, 3]. To address this, several senolytic agents have been used to mitigate cellular consequences of senescence [73, 74]. Both normal aging and AD are characterized by changes to the cholinergic system as reflected by reduction in the levels of acetylcholine, ChAT, nAChRs, mAChRs, and AChE [13, 31, 36], and an increase [32, 36, 38] or no change [37] to the levels of BChE. AChE and BChE are both associated with AD Aβ plaques and tau neurofibrillary tangles [37, 39, 40]. The present work was undertaken to determine whether compounds used as senolytics or nootropics will inhibit ChEs, as these compounds display the clinical benefits of reducing senescent cells, SASP factors or lipofuscin and improving cognitive functions [73, 76–79, 81, 82]. It was reasoned that this knowledge might facilitate the development of next-generation ChEIs/senolytics to treat AD. We focused on evaluating the effects of senolytic and nootropic agents 1-6 on ChEs associated with Aβ plaques and normal neural elements in human AD brain tissues, as well as evaluating the kinetic and *in silico* profiles of compounds to gain insights into their mechanisms of action.

Results from the experimental analyses undertaken showed that compounds 1-6 are ChE inhibitors, with most compounds showing inhibition of both ChEs using Ellman’s method, with *K*_i_ values ranging from 7-110 μM for AChE and 3-95 μM for BChE. In enzyme kinetic studies, compounds showed mixed non-competitive inhibition, often indicative of allosteric binding [105], apart from fisetin (3) which was a competitive inhibitor of AChE and an uncompetitive inhibitor of BChE. The potency of senolytic and nootropic agents 1-6 for ChEs is comparable to the known clinical ChE inhibitors that were previously investigated in *in vitro* and *in situ* studies [37]. For example, galantamine, a ChEI used in the symptomatic treatment of AD, showed inhibition of human AChE and BChE in the μM to sub-μM *K*_i_ range (0.52 μM for AChE, 2.09 μM for BChE) [37], indicating that senolytic and nootropic ChE inhibition is within pharmacological range to warrant future drug development of novel ChEIs based on senolytic and nootropic agents.

Questions about the mechanistic behavior of compounds 1-6 towards ChEs arose in relation to their behavior in histochemical studies. While it is relevant that compounds 1-6 inhibited ChEs in enzyme kinetic studies, it is known that the biochemical properties of AChE and BChE are altered when they are bound to AD pathology compared to when they are associated with normal neural structures [37, 44–46]. This is emphasized by the finding that compounds 1, 2, 5, and 6 selectively inhibit the histochemical staining of both ChEs associated with Aβ plaques but not those associated with normal neural structures. Further, it is of note that the AD drug galantamine inhibits ChE staining associated with both plaques and normal neural elements [37]. The selective, differential inhibition pattern of ChEs with some senolytics is a unique feature that will be desirable in the development of next-generation AD-modifying drugs that solely target pathology-associated ChEs. The selective inhibition of ChEs is consistent with a previous study that observed no effect on AChE activity in the brains of middle-aged and aged female wild-type mice when treated with a cocktail of compounds 1 and 3 [106]. As the KR histochemical method requires potassium ferricyanide and copper sulfate, we found that compounds 3 and 4 most likely chelated with these metals, precluding staining.

Molecular modeling studies were undertaken to determine potential compound binding interactions with ChEs and to help gain insight into their mechanisms of action. Overall, binding studies showed that all compounds have the propensity to bind to ChEs, with some of those being at the active site gorge and others at alternate enzyme binding sites that may represent allosteric binding pockets. Allosteric binding is of relevance as it remains to be determined which region(s) of AChE and BChE bind to Aβ plaques, and allosteric inhibitors may provide insight to this question. Previous studies have suggested that the PAS is the one of the key locations for Aβ binding with AChE [107–109] and BChE [110], and ChE association with Aβ can produce inhibitory or activating effects on ChE catalytic function [110, 111]. Given the observation that plaque-bound ChEs but not normal neural-associated ChEs are inhibited by senolytic and nootropic agents in AD brain tissues, it is likely that the affinity of these compounds for ChE binding sites is increased when Aβ plaques are bound, either through binding to the active site gorge or allosteric sites. The major alternate binding sites on ChEs identified in molecular modeling studies (binding sites II and III; Figure 10) contain key amino acid residues and structural features that are already identified as important for substrate binding and catalysis. At site II this is the ABP and acyl loop [94, 112–116], while at site III this is the catalytic glutamate and Y332 (Y341 for BChE), which are important residues of the ε-helix [117–119]. Therefore, it is plausible that compound binding in or near these sites would alter catalytic activity or inhibit catalysis altogether. Generally, *in silico* docking studies showed that there are four main putative allosteric sites on ChEs that compounds 1-6 may be binding to produce inhibitory activity. As these sites are of relevance to both inhibition of activity and Aβ interactions [110, 111], they could be utilized as targets for the development of future ChEIs.

There are several limitations within the current study. This includes a broader, qualitative analysis of a relatively small sample size of sex- and age-matched human AD brain tissues, which precluded us from statistical analysis and examining the potential impact of sex, age and plaque load on the senolytic and nootropic inhibition of ChEs associated with AD pathology. The incidence of AD increases with age and this neurodegenerative disorder is more prevalent in females than males, not accounted for by longer life expectancy in females [120]. Studies have demonstrated that higher AD neuropathology is attendant with greater cognitive decline in females [121, 122]. Moreover, it has been shown that when BChE is knocked-out in the 5XFAD mouse model of amyloidosis, there is a significantly lower percentage of fibrillar Aβ plaques in cortical and subcortical regions than in the parent 5XFAD strain, an effect more pronounced in males [123, 124]. This finding makes the relationship of sex, age and ChE-related AD pathology to be a point of interest, particularly in the context of senolytic and nootropic inhibition. A larger sex and age-matched sample size would allow for identifying such differences, if any, and help facilitate development of disease-modifying senolytic or nootropic drugs that have sex-specific targets towards personalized medicine.

Previous studies in the senescence-accelerated prone 8 (SAMP8) mouse, a model of aging that recapitulates many of the cognitive and neuropathological characteristics of AD [125, 126], showed that AChE activity is unchanged while BChE activity is 2-fold higher in the brain when compared to senescent-resistant control mice [22, 23]. AChE and BChE expression in the SAMP8 mouse brain was unchanged [22, 23] or not significantly changed [18] compared to control mice, with the exception of significantly increased BChE expression in the SAMP8 hippocampus [18]. The upregulation of BChE activity in the SAMP8 model is in accordance with similar findings in human AD brains [38, 41] and in AD mouse models of amyloidosis [123, 127]. The increase in activity but not expression of BChE in SAMP8 mice was primarily attributed to the increased abundance and phenotypic changes of neuroglia [22, 23], a major source of brain BChE [28, 128–132]. The upregulated proliferation and activation of neuroglia, as well as their sustained release of pro-inflammatory cytokines [22, 23], are characteristic hallmarks of SAMP8 mouse brains [133, 134], as well as human age-related senescent and AD brains [2].

Since the increasing accumulation of Aβ plaque deposits and senescent cells are capable of increasing inflammation through secretion of pro-inflammatory cytokines, the CAIP becomes important in modulating anti-inflammatory activities. In this pathway, there is increased inflammation with low levels of acetylcholine leading to the secretion of pro-inflammatory cytokines such as TNF-α, IL-1β, and IL-6; however, in the presence of appropriate or increased levels of acetylcholine there is protection against inflammation via suppression of secreted pro-inflammatory cytokines [66, 67, 69, 72]. Due to the close, intrinsic relationship between BChE and neuroinflammation in age-related senescence and AD, there is justification for the use of BChE inhibitors [70, 71] to increase the availability of acetylcholine thereby modulating anti-inflammatory effects via the CAIP [61, 133, 135]. In this regard, all the senolytic and nootropic compounds evaluated herein are inhibitors of ChEs, particularly AChE and BChE that are bound to AD pathology. Although the anti-inflammatory effects of 1-6 could not be investigated in the post-mortem tissue model employed herein, it could be inferred that any possible anti-inflammatory effects of these compounds, produced through the inhibition of SASP factors, may be, in part, through modulation of the CAIP [71]. However, there may be other pathways that are also involved [136], including allosteric sites that could be involved in pre-empting association of these enzymes to AD pathology. Future work investigating the anti-inflammatory effect of compounds 1-6 on the CAIP or other pathways in *in vivo* or *in vitro* models will require further investigations.

In conclusion, the senolytic compounds evaluated herein may have beneficial effects on both aging and AD, at least in part, through modulation of AChE and BChE associated with AD pathology. This work provides new opportunities for the development of the next generation of ChE inhibitors that specifically target AChE and BChE associated with AD pathology.

## MATERIALS AND METHODS

### Materials

Acetonitrile was purchased from Fisher Scientific (Ottawa, Ontario, Canada). Acetylthiocholine iodide (ATChI), butyrylthiocholine iodide (BTChI), BW 284c51, cobalt chloride, cupric sulfate, 3,3’-diaminobenzidine tetrahydrochloride (DAB), 5,5-dithio-bis-(2-nitrobenzoic acid) (DTNB), ethanol, ethopropazine, gelatin, potassium ferricyanide, recombinant human AChE, and sodium citrate were purchased from MilliporeSigma Canada Ltd. (Oakville, Ontario, Canada). Dasatinib, nintedanib (Bibfl120), fisentin, quercetin, and GW2580 were purchased from Adooq Biosciences (Irvine, CA, USA). Meclofenoxate hydrochloride was purchased from Combi-Blocks (San Diego, CA, USA). All chemicals were used as received from suppliers. BChE purified from human plasma was a gift from Dr. Oksana Lockridge (Eppley Institute, University of Nebraska Medical Center, Omaha, NE, USA). Enzyme kinetic experiments were performed using a VWR UV-1600PC spectrophotometer (VWR International LLC, Canada, Mississauga, Ontario, Canada) with M.Wave Professional software 1.0.20 (Azzota Scientific, DE, USA). All enzyme kinetic data were analyzed, and Lineweaver-Burk plots were generated for each enzyme kinetic experiment using Microsoft Excel for Microsoft 365 MSO (version 2409 build 18025.20104; Microsoft Corporation, Redmond, WA, USA). Plots were assembled into figures using Adobe Photoshop (CS 5, Version 12.0, San Diego, CA, USA).

### Enzyme kinetic studies

Enzyme kinetic studies were performed as previously described [86], using Ellman’s assay [137]. Briefly, in a quartz cuvette (1 cm pathlength), a 0.577 mM DTNB solution in 0.1 M potassium phosphate buffer (1.6 mL, pH 7.0) was combined at room temperature with either recombinant AChE (0.1 mL, 3.7 nM) in 0.005% aqueous gelatin or purified human serum BChE (0.1 mL, 5.85 nM) in 0.005% aqueous gelatin and mixed with a senolytic or nootropic compound (Figure 1) dissolved in 50% (v/v) aqueous acetonitrile (0.05 mL). Absorbance of the cuvette solution was measured at 412 nm and the instrument zeroed before initiation of the reaction with either the AChE substrate ATChI (0.05 mL, 166 μM) or the BChE substrate BTChI (0.05 mL, 166 μM), dissolved in water. Substrate concentrations were kept constant while the senolytic concentrations varied (0-140 μM). Changes in absorbance (ΔA/min), reflecting the rate of ATChI or BTChI hydrolysis with their respective enzymes, were recorded spectrophotometrically every 5 seconds for 1 minute.

As done previously [86], Lineweaver-Burk plots were generated for each senolytic and nootropic compound with the velocity (v) of each reaction determined using Beer’s Law, where v = (ΔA/min)/(εl), ε is the molar extinction coefficient for 5-thio-2-nitrobenzoic acid (TNB) (13600 M^−1^ cm^−1^), and l is the cuvette pathlength (1 cm). Replotting the calculated slopes from each double reciprocal plot against senolytic and nootropic concentrations, gave the inhibitor constant (*K*_i_, M) as the x-intercept. The mode of inhibition (competitive, non-competitive, mixed non-competitive, or uncompetitive) was also determined by analyzing the relational pattern of linear regressions for varying concentrations of the same senolytic or nootropic compound. Enzyme kinetic experiments were performed in triplicate and kinetic parameter values were averaged.

### Brain tissues

Human orbitofrontal cortex and thalamic brain tissues were used to evaluate the effects of senolytic and nootropic compounds on brain ChE activity associated with Aβ plaques and normal neural elements (i.e. neurons, neuropil, and axons), respectively. Brain regions were chosen based on the known distribution of AChE and BChE associated with normal neural elements (i.e. thalamus) and Aβ plaques (i.e. orbitofrontal cortex), as done previously [41, 138]. Following approval from the Nova Scotia Health Research Ethics Board, post-mortem brain tissues from sex- and age-matched AD cases that fulfilled clinical [139] and neuropathological [140–142] criteria for AD were obtained from the Maritime Brain Tissue Bank, a Centralized Operation of Research Equipment and Supports (CORES) facility, Faculty of Medicine, Dalhousie University (Halifax, Nova Scotia, Canada). Demographic information related to the cases used in this study can be found in Table 2.

The brains were removed between 10-23 hr after death and bisected at the midline. One half of the brain was sent for neuropathological examination, and the other was used for histochemical studies. The tissues used for histochemical studies were cut into approximately 1 cm-thick coronal slabs and immersion fixed in 10% buffered formalin between 2.11-4 days. Slabs were cryoprotected in a graded series of sucrose (10-40%) in 0.1 M phosphate buffer (pH 7.4; PB) for a minimum of 48 hr per sucrose solution and stored in PB with 40% sucrose and 0.6% sodium azide. Orbitofrontal and thalamic regions were sub-sectioned from the slabs and tissue blocks were cut into 50 μM-thick serial sections using a Leica SM2000R microtome with a Physitemp freezing stage and BFS-40MPA controller (Physitemp Instruments LLC, Clifton, NJ, USA). Tissue sections were stored at -20° C in in PB with 40% sucrose and 0.05% sodium azide until used.

### Histochemical studies

Histochemical staining for AChE and BChE activity in brain tissues was done using a modified [42] Karnovsky-Roots (KR) method [143]. Tissue sections were first rinsed for 30 min in 0.1 M PB, followed by 30 min in 0.1 M PB with 15% hydrogen peroxide (H_2_O_2_) and then rinsed for 30 min with 0.1 M maleate buffer (MB; pH 6.8 or 7.4) prior to incubation in the KR solution. Sections stained for AChE were incubated in the KR solution for 1.75 hr, while sections stained for BChE were incubated for 2.5 hr. The KR staining solution contained 0.5 mM sodium citrate, 0.47 mM cupric sulfate, and 0.05 mM of potassium ferricyanide, a thioester ChE substrate and ChE inhibitor in MB at pH 6.8 to stain for ChEs associated with AD pathology or pH 8.0 to stain for ChEs associated with normal neural elements. For AChE staining, ATChI (0.4 mM) was used as the substrate and ethopropazine (0.06 mM) was used to inhibit BChE. For BChE staining, BTChI (0.8 mM) was used as the substrate and BW 284c51 (0.01 mM) was used to inhibit AChE. Following incubation in KR, sections were rinsed in distilled water (dH_2_O) for 30 min, incubated in a 0.1% cobalt chloride solution in dH_2_O for 10 min, and rinsed again in dH_2_0 for 30 min. Sections were incubated in 1.39 mM 3,3’-diaminobenzidine tetrahydrochloride (DAB) in PB (pH 7.4) for 5 min and then developed using 50 μL of 0.3% H_2_O_2_ per mL of DAB solution. The reaction was stopped by a 30 min rinse in 0.01 M acetate buffer (pH 3.3). Stained sections were mounted on glass slides, cover-slipped and examined with brightfield microscopy.

To evaluate the interactions of senolytic and nootropic compounds with ChEs in brain tissues, the above histochemical technique was used with minor variations to the KR incubation [88]. Senolytic and nootropic compounds were dissolved in 50% aqueous acetonitrile to make a stock solution. The same volume of each compound was added individually to the KR solution; 0.25 mM dasatinib (1); 0.27 mM nintedanib (2); 1.3 mM fisetin (3); 1.3 mM quercetin (4); 0.4 mM GW2580 (5); and 1.3 mM meclofenoxate hydrochloride (6). Following incubation for AChE (1.75 hr) and BChE (2.5 hr) in the KR solution, staining was completed as described above.

To ensure specificity of staining and that changes in staining were due to the presence of compounds 1-6, control experiments were conducted. Positive controls included staining for BChE and AChE activity at pH 6.8 and pH 8.0 without any additions. Negative controls were utilized to validate staining specificity of BChE and AChE at pH 6.8 and pH 8.0, where the substrates (BTChI and ATChI, respectively) were omitted, as previously described [88]. When the substrates were omitted, no BChE or AChE staining was observed. An additional control experiment was used to test the effect of the carrier solvent for compounds 1-6 (50% aqueous acetonitrile) on KR staining. Briefly, 50% aqueous acetonitrile was added to the KR solution, without any senolytic or nootropic compound, while maintaining the same volume as the compound additives. When 50% aqueous acetonitrile was used in KR staining, background staining intensity increased for AChE pH 6.8, while both AChE and BChE pH 6.8 plaques showed some reduction in overall staining. The extent of reduction by carrier solvent did not preclude evaluation of the inhibitory effects of compounds 1-6 on ChE histochemical staining. There were no observed effects on normal neural elements at pH 8.0.

A qualitative method was used to evaluate the effect of senolytic and nootropic compounds on ChE histochemical staining, as done previously [88]. Inhibition of staining intensity was categorized as follows: No reduction (-), slight (x), moderate (xx), or strong (xxx). Tissue sections were analyzed using brightfield microscopy on an Olympus BX50F microscope (Olympus Optical Co., Ltd., Tokyo, Japan). Photomicrographs of the stained tissue sections were taken using a Zeiss Axio Scan.Z1 slide scanner with Zen 3.1 Blue Edition software (Carl Zeiss Canada Ltd., Toronto, Ontario, Canada). Figures were assembled using Adobe Photoshop (CS 5, Version 12.0, San Diego, CA, USA). The brightness of each individual image was adjusted to ensure backgrounds matched for analysis.

### Molecular docking studies

To determine predicted binding sites for the senolytic and nootropic agents with human AChE and BChE, compounds were docked using a reversible inhibitor docking procedure with Molecular Operating Environment (MOE) software 2022.02 (Chemical Computing Group, Montreal, Canada), as previously described [144, 145]. This docking procedure consisted of a blind docking phase, in which all compounds were allowed to dock anywhere on the enzyme, and a site-directed docking phase, in which compounds were directed to dock to their respective enzyme active site or other sites identified through blind docking. Blind docks were conducted to determine whether a compound was likely to find the active site of a given enzyme or if other enzyme locations, such as allosteric sites, were also favorable for compound binding. Site-directed docks were conducted to determine possible key interactions between the compound and enzyme residues and to predict inhibition constants (*K*_i_ values).

Crystal structures for human AChE (Protein Databank code (PDB): 4M0E, 2.00 Å resolution) [91] and BChE (PDB: 4TPK, 2.70 Å) [92] were obtained from the Protein Databank [146] and were selected based on resolution and co-crystallization properties deemed appropriate for molecular docking [100]. Crystal structures were prepared for molecular docking as previously described [144] with all water molecules removed. Enzyme sites from blind docks were analyzed to identify locations for subsequent site-directed docks. The binding energies of the top five returned site-directed dock poses, key amino acid interactions, and bond lengths were identified and measured within MOE. Docks were completed in triplicate and their binding energies were converted to predicted *K*_i_ values using Gibb’s free energy equation and averaged, as done previously [147].

## Supplementary Material

Supplementary Figure 1

Supplementary Tables

## References

[1] Hayflick L, Moorhead PS. The serial cultivation of human diploid cell strains. Exp Cell Res. 1961; 25:585–621. 10.1016/0014-4827(61)90192-6 13905658

[2] Saez-Atienzar S, Masliah E. Cellular senescence and Alzheimer disease: the egg and the chicken scenario. Nat Rev Neurosci. 2020; 21:433–44. 10.1038/s41583-020-0325-z 32601397 PMC12548380

[3] Holloway K, Neherin K, Dam KU, Zhang H. Cellular senescence and neurodegeneration. Hum Genet. 2023; 142:1247–62. 10.1007/s00439-023-02565-x 37115318

[4] Engeland K. Cell cycle regulation: p53-p21-RB signaling. Cell Death Differ. 2022; 29:946–60. 10.1038/s41418-022-00988-z 35361964 PMC9090780

[5] Safwan-Zaiter H, Wagner N, Wagner KD. P16INK4A-More Than a Senescence Marker. Life (Basel). 2022; 12:1332. 10.3390/life12091332 36143369 PMC9501954

[6] Dimri GP, Lee X, Basile G, Acosta M, Scott G, Roskelley C, Medrano EE, Linskens M, Rubelj I, Pereira-Smith O. A biomarker that identifies senescent human cells in culture and in aging skin *in vivo*. Proc Natl Acad Sci USA. 1995; 92:9363–7. 10.1073/pnas.92.20.9363 7568133 PMC40985

[7] Georgakopoulou EA, Tsimaratou K, Evangelou K, Fernandez Marcos PJ, Zoumpourlis V, Trougakos IP, Kletsas D, Bartek J, Serrano M, Gorgoulis VG. Specific lipofuscin staining as a novel biomarker to detect replicative and stress-induced senescence. A method applicable in cryo-preserved and archival tissues. Aging (Albany NY). 2013; 5:37–50. 10.18632/aging.100527 23449538 PMC3616230

[8] Baker DJ, Petersen RC. Cellular senescence in brain aging and neurodegenerative diseases: evidence and perspectives. J Clin Invest. 2018; 128:1208–16. 10.1172/JCI95145 29457783 PMC5873891

[9] Di Micco R, Krizhanovsky V, Baker D, d’Adda di Fagagna F. Cellular senescence in ageing: from mechanisms to therapeutic opportunities. Nat Rev Mol Cell Biol. 2021; 22:75–95. 10.1038/s41580-020-00314-w 33328614 PMC8344376

[10] Jurk D, Wang C, Miwa S, Maddick M, Korolchuk V, Tsolou A, Gonos ES, Thrasivoulou C, Saffrey MJ, Cameron K, von Zglinicki T. Postmitotic neurons develop a p21-dependent senescence-like phenotype driven by a DNA damage response. Aging Cell. 2012; 11:996–1004. 10.1111/j.1474-9726.2012.00870.x 22882466 PMC3533793

[11] Gallagher M, Colombo PJ. Ageing: the cholinergic hypothesis of cognitive decline. Curr Opin Neurobiol. 1995; 5:161–8. 10.1016/0959-4388(95)80022-0 7620303

[12] Orlando IF, Shine JM, Robbins TW, Rowe JB, O’Callaghan C. Noradrenergic and cholinergic systems take centre stage in neuropsychiatric diseases of ageing. Neurosci Biobehav Rev. 2023; 149:105167. 10.1016/j.neubiorev.2023.105167 37054802

[13] Schliebs R, Arendt T. The cholinergic system in aging and neuronal degeneration. Behav Brain Res. 2011; 221:555–63. 10.1016/j.bbr.2010.11.058 21145918

[14] Gibson GE, Peterson C, Jenden DJ. Brain acetylcholine synthesis declines with senescence. Science. 1981; 213:674–6. 10.1126/science.7256270 7256270

[15] Ikegami S, Shumiya S, Kawamura H. Age-related changes in radial-arm maze learning and basal forebrain cholinergic systems in senescence accelerated mice (SAM). Behav Brain Res. 1992; 51:15–22. 10.1016/s0166-4328(05)80307-9 1482543

[16] Perry EK, Piggott MA, Court JA, Johnson M, Perry RH. Transmitters in the developing and senescent human brain. Ann N Y Acad Sci. 1993; 695:69–72. 10.1111/j.1749-6632.1993.tb23030.x 7902056

[17] Liu D, Hsueh SC, Tweedie D, Price N, Glotfelty E, Lecca D, Telljohann R, deCabo R, Hoffer BJ, Greig NH. Chronic inflammation with microglia senescence at basal forebrain: impact on cholinergic deficit in Alzheimer’s brain haemodynamics. Brain Commun. 2024; 6:fcae204. 10.1093/braincomms/fcae204 38978722 PMC11228546

[18] Reale M, Costantini E, Aielli L, Di Giuseppe F, Angelucci S, Kamal MA, Greig NH. Proteomic Signature and mRNA Expression in Hippocampus of SAMP8 and SAMR1 Mice during Aging. Int J Mol Sci. 2022; 23:15097. 10.3390/ijms232315097 36499421 PMC9740614

[19] Vijayan VK. Cholinergic enzymes in the cerebellum and the hippocampus of the senescent mouse. Exp Gerontol. 1977; 12:7–11. 10.1016/0531-5565(77)90026-2 885175

[20] Kabuto H, Yokoi I, Mori A, Murakami M, Sawada S. Neurochemical changes related to ageing in the senescence-accelerated mouse brain and the effect of chronic administration of nimodipine. Mech Ageing Dev. 1995; 80:1–9. 10.1016/0047-6374(94)01542-t 7564556

[21] Nitta A, Naruhashi K, Umemura M, Hasegawa T, Furukawa S, Sekiguchi F, Ishibashi K, Nabeshima T. Age-related changes in learning and memory and cholinergic neuronal function in senescence accelerated mice (SAM). Behav Brain Res. 1995; 72:49–55. 10.1016/0166-4328(96)00040-x 8788856

[22] Fernández-Gómez FJ, Muñoz-Delgado E, Montenegro MF, Campoy FJ, Vidal CJ, Jordán J. The level of butyrylcholinesterase activity increases and the content of the mRNA remains unaffected in brain of senescence-accelerated mouse SAMP8. Chem Biol Interact. 2008; 175:332–5. 10.1016/j.cbi.2008.05.010 18571151

[23] Fernández-Gómez FJ, Muñoz-Delgado E, Montenegro MF, Campoy FJ, Vidal CJ, Jordán J. Cholinesterase activity in brain of senescence-accelerated-resistant mouse SAMR1 and its variation in brain of senescence-accelerated-prone mouse SAMP8. J Neurosci Res. 2010; 88:155–66. 10.1002/jnr.22177 19610099

[24] Darvesh S, Hopkins DA, Geula C. Neurobiology of butyrylcholinesterase. Nat Rev Neurosci. 2003; 4:131–8. 10.1038/nrn1035 12563284

[25] Lockridge O. Review of human butyrylcholinesterase structure, function, genetic variants, history of use in the clinic, and potential therapeutic uses. Pharmacol Ther. 2015; 148:34–46. 10.1016/j.pharmthera.2014.11.011 25448037

[26] Darvesh S, Grantham DL, Hopkins DA. Distribution of butyrylcholinesterase in the human amygdala and hippocampal formation. J Comp Neurol. 1998; 393:374–90. 9548556

[27] Friede RL. A comparative histochemical mapping of the distribution of butyryl cholinesterase in the brains of four species of mammals, including man. Acta Anat (Basel). 1967; 66:161–77. 10.1159/000142920 4964503

[28] Silver A. The Biology of Cholinesterases. (Amsterdam, Netherlands: North-Holland Publishing Company). 1974.

[29] Massoulié J, Sussman J, Bon S, Silman I. Structure and functions of acetylcholinesterase and butyrylcholinesterase. Prog Brain Res. 1993; 98:139–46. 10.1016/s0079-6123(08)62391-2 8248501

[30] Villeda-González JD, Gómez-Olivares JL, Baiza-Gutman LA. New paradigms in the study of the cholinergic system and metabolic diseases: Acetyl-and-butyrylcholinesterase. J Cell Physiol. 2024; 239:e31274. 10.1002/jcp.31274 38605655

[31] Pepeu G, Giovannelli L. The central cholinergic system during aging. Prog Brain Res. 1994; 100:67–71. 10.1016/s0079-6123(08)60770-0 7938536

[32] Perry EK. The cholinergic system in old age and Alzheimer’s disease. Age Ageing. 1980; 9:1–8. 10.1093/ageing/9.1.1 7361631

[33] Chaves-Coira I, García-Magro N, Zegarra-Valdivia J, Torres-Alemán I, Núñez Á. Cognitive Deficits in Aging Related to Changes in Basal Forebrain Neuronal Activity. Cells. 2023; 12:1477. 10.3390/cells12111477 37296598 PMC10252596

[34] Bartus RT, Dean RL 3rd, Beer B, Lippa AS. The cholinergic hypothesis of geriatric memory dysfunction. Science. 1982; 217:408–14. 10.1126/science.7046051 7046051

[35] Coyle JT, Price DL, DeLong MR. Alzheimer’s disease: a disorder of cortical cholinergic innervation. Science. 1983; 219:1184–90. 10.1126/science.6338589 6338589

[36] Perry EK, Perry RH, Blessed G, Tomlinson BE. Changes in brain cholinesterases in senile dementia of Alzheimer type. Neuropathol Appl Neurobiol. 1978; 4:273–7. 10.1111/j.1365-2990.1978.tb00545.x 703927

[37] Darvesh S, Reid GA, Martin E. Biochemical and histochemical comparison of cholinesterases in normal and Alzheimer brain tissues. Curr Alzheimer Res. 2010; 7:386–400. 10.2174/156720510791383868 19939227

[38] Atack JR, Perry EK, Bonham JR, Candy JM, Perry RH. Molecular forms of butyrylcholinesterase in the human neocortex during development and degeneration of the cortical cholinergic system. J Neurochem. 1987; 48:1687–92. 10.1111/j.1471-4159.1987.tb05724.x 3572398

[39] Mesulam MM, Geula C. Butyrylcholinesterase reactivity differentiates the amyloid plaques of aging from those of dementia. Ann Neurol. 1994; 36:722–7. 10.1002/ana.410360506 7979218

[40] Geula C, Mesulam MM. Cholinesterases and the pathology of Alzheimer disease. Alzheimer Dis Assoc Disord. 1995; 9:23–8. 10.1097/00002093-199501002-00005 8534419

[41] Macdonald IR, Maxwell SP, Reid GA, Cash MK, DeBay DR, Darvesh S. Quantification of Butyrylcholinesterase Activity as a Sensitive and Specific Biomarker of Alzheimer’s Disease. J Alzheimers Dis. 2017; 58:491–505. 10.3233/JAD-170164 28453492 PMC5438481

[42] Maxwell SP, Cash MK, Darvesh S. Neuropathology and cholinesterase expression in the brains of octogenarians and older. Chem Biol Interact. 2022; 364:110065. 10.1016/j.cbi.2022.110065 35872043

[43] Maxwell SPL. Clinicopathological Correlates in Tauopathy and Aging. Medical Neurosience. (Halifax, Canada: Dalhousie University). 2022; 1–197.

[44] Geula C, Mesulam M. Special properties of cholinesterases in the cerebral cortex of Alzheimer’s disease. Brain Res. 1989; 498:185–9. 10.1016/0006-8993(89)90419-8 2790472

[45] Reid GA, Darvesh S. Interaction of Exogenous Butyrylcholinesterase with β-Amyloid Plaques in 5XFAD/Butyrylcholinesterase-Knockout Mouse Brain. Curr Alzheimer Res. 2021; 18:470–81. 10.2174/1567205018666210827122704 34455970

[46] Reid GA, Darvesh S. Interaction of exogenous acetylcholinesterase and butyrylcholinesterase with amyloid-β plaques in human brain tissue. Chem Biol Interact. 2024; 395:111012. 10.1016/j.cbi.2024.111012 38648920

[47] Wright CI, Geula C, Mesulam MM. Neurological cholinesterases in the normal brain and in Alzheimer’s disease: relationship to plaques, tangles, and patterns of selective vulnerability. Ann Neurol. 1993; 34:373–84. 10.1002/ana.410340312 8363355

[48] Gok M, Cicek C, Bodur E. Butyrylcholinesterase in lipid metabolism: A new outlook. J Neurochem. 2024; 168:381–5. 10.1111/jnc.15833 37129444

[49] Jasiecki J, Szczoczarz A, Cysewski D, Lewandowski K, Skowron P, Waleron K, Wasąg B. Butyrylcholinesterase-Protein Interactions in Human Serum. Int J Mol Sci. 2021; 22:10662. 10.3390/ijms221910662 34639003 PMC8508650

[50] Paraoanu LE, Steinert G, Klaczinski J, Becker-Röck M, Bytyqi A, Layer PG. On functions of cholinesterases during embryonic development. J Mol Neurosci. 2006; 30:201–4. 10.1385/JMN:30:1:201 17192676

[51] Vidal CJ. Expression of cholinesterases in brain and non-brain tumours. Chem Biol Interact. 2005; 157–158:227–32. 10.1016/j.cbi.2005.10.035 16256970

[52] Johnson G, Moore SW. Cholinesterases modulate cell adhesion in human neuroblastoma cells *in vitro*. Int J Dev Neurosci. 2000; 18:781–90. 10.1016/s0736-5748(00)00049-6 11154847

[53] Comoletti D, Trobiani L, Chatonnet A, Bourne Y, Marchot P. Comparative mapping of selected structural determinants on the extracellular domains of cholinesterase-like cell-adhesion molecules. Neuropharmacology. 2021; 184:108381. 10.1016/j.neuropharm.2020.108381 33166544

[54] Barbosa M, Rios O, Velásquez M, Villalobos J, Ehrmanns J. Acetylcholinesterase and butyrylcholinesterase histochemical activities and tumor cell growth in several brain tumors. Surg Neurol. 2001; 55:106–12. 10.1016/s0090-3019(01)00351-2 11301094

[55] Baranowska-Kortylewicz J, Kortylewicz ZP, McIntyre EM, Sharp JG, Coulter DW. Multifarious Functions of Butyrylcholinesterase in Neuroblastoma: Impact of BCHE Deletion on the Neuroblastoma Growth *In Vitro* and *In Vivo*. J Pediatr Hematol Oncol. 2022; 44:293–304. 10.1097/MPH.0000000000002285 34486544

[56] Mack A, Robitzki A. The key role of butyrylcholinesterase during neurogenesis and neural disorders: an antisense-5'butyrylcholinesterase-DNA study. Prog Neurobiol. 2000; 60:607–28. 10.1016/s0301-0082(99)00047-7 10739090

[57] Carneiro BA, El-Deiry WS. Targeting apoptosis in cancer therapy. Nat Rev Clin Oncol. 2020; 17:395–417. 10.1038/s41571-020-0341-y 32203277 PMC8211386

[58] Blagosklonny MV. Anti-aging: senolytics or gerostatics (unconventional view). Oncotarget. 2021; 12:1821–35. 10.18632/oncotarget.28049 34504654 PMC8416555

[59] Skrzypek A, Karpińska M, Juszczak M, Grabarska A, Wietrzyk J, Krajewska-Kułak E, Studziński M, Paszko T, Matysiak J. Cholinesterases Inhibition, Anticancer and Antioxidant Activity of Novel Benzoxazole and Naphthoxazole Analogs. Molecules. 2022; 27:8511. 10.3390/molecules27238511 36500605 PMC9738531

[60] Katalinić M, Rusak G, Domaćinović Barović J, Sinko G, Jelić D, Antolović R, Kovarik Z. Structural aspects of flavonoids as inhibitors of human butyrylcholinesterase. Eur J Med Chem. 2010; 45:186–92. 10.1016/j.ejmech.2009.09.041 19879672

[61] Macdonald IR, Rockwood K, Martin E, Darvesh S. Cholinesterase inhibition in Alzheimer’s disease: is specificity the answer? J Alzheimers Dis. 2014; 42:379–84. 10.3233/JAD-140219 24898642

[62] Birks J. Cholinesterase inhibitors for Alzheimer’s disease. Cochrane Database Syst Rev. 2006; 2006:CD005593. 10.1002/14651858.CD005593 16437532 PMC9006343

[63] Arias E, Gallego-Sandín S, Villarroya M, García AG, López MG. Unequal neuroprotection afforded by the acetylcholinesterase inhibitors galantamine, donepezil, and rivastigmine in SH-SY5Y neuroblastoma cells: role of nicotinic receptors. J Pharmacol Exp Ther. 2005; 315:1346–53. 10.1124/jpet.105.090365 16144975

[64] Gupta P, Tiwari S, Singh A, Pal A, Mishra A, Singh S. Rivastigmine attenuates the Alzheimer’s disease related protein degradation and apoptotic neuronal death signalling. Biochem J. 2021; 478:1435–51. 10.1042/BCJ20200754 33660768

[65] Reale M, Iarlori C, Gambi F, Lucci I, Salvatore M, Gambi D. Acetylcholinesterase inhibitors effects on oncostatin-M, interleukin-1 beta and interleukin-6 release from lymphocytes of Alzheimer’s disease patients. Exp Gerontol. 2005; 40:165–71. 10.1016/j.exger.2004.12.003 15763393

[66] Pavlov VA, Parrish WR, Rosas-Ballina M, Ochani M, Puerta M, Ochani K, Chavan S, Al-Abed Y, Tracey KJ. Brain acetylcholinesterase activity controls systemic cytokine levels through the cholinergic anti-inflammatory pathway. Brain Behav Immun. 2009; 23:41–45. 10.1016/j.bbi.2008.06.011 18639629 PMC4533839

[67] Nizri E, Brenner T. Modulation of inflammatory pathways by the immune cholinergic system. Amino Acids. 2013; 45:73–85. 10.1007/s00726-011-1192-8 22194043

[68] Gamage R, Wagnon I, Rossetti I, Childs R, Niedermayer G, Chesworth R, Gyengesi E. Cholinergic Modulation of Glial Function During Aging and Chronic Neuroinflammation. Front Cell Neurosci. 2020; 14:577912. 10.3389/fncel.2020.577912 33192323 PMC7594524

[69] Borovikova LV, Ivanova S, Zhang M, Yang H, Botchkina GI, Watkins LR, Wang H, Abumrad N, Eaton JW, Tracey KJ. Vagus nerve stimulation attenuates the systemic inflammatory response to endotoxin. Nature. 2000; 405:458–62. 10.1038/35013070 10839541

[70] Liu EY, Xia Y, Kong X, Guo MS, Yu AX, Zheng BZ, Mak S, Xu ML, Tsim KW. Interacting with α 7 nAChR is a new mechanism for AChE to enhance the inflammatory response in macrophages. Acta Pharm Sin B. 2020; 10:1926–42. 10.1016/j.apsb.2020.05.005 33163344 PMC7606108

[71] Moreira NC, Lima JE, Marchiori MF, Carvalho I, Sakamoto-Hojo ET. Neuroprotective Effects of Cholinesterase Inhibitors: Current Scenario in Therapies for Alzheimer’s Disease and Future Perspectives. J Alzheimers Dis Rep. 2022; 6:177–93. 10.3233/ADR-210061 35591949 PMC9108627

[72] Benfante R, Di Lascio S, Cardani S, Fornasari D. Acetylcholinesterase inhibitors targeting the cholinergic anti-inflammatory pathway: a new therapeutic perspective in aging-related disorders. Aging Clin Exp Res. 2021; 33:823–34. 10.1007/s40520-019-01359-4 31583530

[73] Raffaele M, Vinciguerra M. The costs and benefits of senotherapeutics for human health. Lancet Healthy Longev. 2022; 3:e67–77. 10.1016/S2666-7568(21)00300-7 36098323

[74] Riessland M, Orr ME. Translating the Biology of Aging into New Therapeutics for Alzheimer’s Disease: Senolytics. J Prev Alzheimers Dis. 2023; 10:633–46. 10.14283/jpad.2023.104 37874084 PMC11103249

[75] Zs-Nagy I. Pharmacological interventions against aging through the cell plasma membrane: a review of the experimental results obtained in animals and humans. Ann N Y Acad Sci. 2002. 10.1111/j.1749-6632.2002.tb02102.x 11976205

[76] Bussian TJ, Aziz A, Meyer CF, Swenson BL, van Deursen JM, Baker DJ. Clearance of senescent glial cells prevents tau-dependent pathology and cognitive decline. Nature. 2018; 562:578–82. 10.1038/s41586-018-0543-y 30232451 PMC6206507

[77] Musi N, Valentine JM, Sickora KR, Baeuerle E, Thompson CS, Shen Q, Orr ME. Tau protein aggregation is associated with cellular senescence in the brain. Aging Cell. 2018; 17:e12840. 10.1111/acel.12840 30126037 PMC6260915

[78] Zhang P, Kishimoto Y, Grammatikakis I, Gottimukkala K, Cutler RG, Zhang S, Abdelmohsen K, Bohr VA, Misra Sen J, Gorospe M, Mattson MP. Senolytic therapy alleviates Aβ-associated oligodendrocyte progenitor cell senescence and cognitive deficits in an Alzheimer’s disease model. Nat Neurosci. 2019; 22:719–28. 10.1038/s41593-019-0372-9 30936558 PMC6605052

[79] Hu Y, Fryatt GL, Ghorbani M, Obst J, Menassa DA, Martin-Estebane M, Muntslag TA, Olmos-Alonso A, Guerrero-Carrasco M, Thomas D, Cragg MS, Gomez-Nicola D. Replicative senescence dictates the emergence of disease-associated microglia and contributes to Aβ pathology. Cell Rep. 2021; 35:109228. 10.1016/j.celrep.2021.109228 34107254 PMC8206957

[80] Zhu Y, Tchkonia T, Fuhrmann-Stroissnigg H, Dai HM, Ling YY, Stout MB, Pirtskhalava T, Giorgadze N, Johnson KO, Giles CB, Wren JD, Niedernhofer LJ, Robbins PD, Kirkland JL. Identification of a novel senolytic agent, navitoclax, targeting the Bcl-2 family of anti-apoptotic factors. Aging Cell. 2016; 15:428–35. 10.1111/acel.12445 26711051 PMC4854923

[81] Chaib S, Tchkonia T, Kirkland JL. Cellular senescence and senolytics: the path to the clinic. Nat Med. 2022; 28:1556–68. 10.1038/s41591-022-01923-y 35953721 PMC9599677

[82] Nandy K, Bourne GH. Effect of centrophenoxine on the lipofuscin pigments in the neurones of senile guinea-pigs. Nature. 1966; 210:313–4. 10.1038/210313a0 4380940

[83] Nandy K. Centrophenoxine: effects on aging mammalian brain. J Am Geriatr Soc. 1978; 26:74–81. 10.1111/j.1532-5415.1978.tb02544.x 342588

[84] Bhalla P, Nehru B. Modulatory effects of centrophenoxine on different regions of ageing rat brain. Exp Gerontol. 2005; 40:801–6. 10.1016/j.exger.2005.06.016 16137852

[85] Pohanka M. Inhibitors of acetylcholinesterase and butyrylcholinesterase meet immunity. Int J Mol Sci. 2014; 15:9809–25. 10.3390/ijms15069809 24893223 PMC4100123

[86] Sands D, Davis A, Banfield S, Pottie IR, Darvesh S. Solvents and detergents compatible with enzyme kinetic studies of cholinesterases. Chem Biol Interact. 2023; 383:110667. 10.1016/j.cbi.2023.110667 37579937

[87] Orhan I, Kartal M, Tosun F, Sener B. Screening of various phenolic acids and flavonoid derivatives for their anticholinesterase potential. Z Naturforsch C J Biosci. 2007; 62:829–32. 10.1515/znc-2007-11-1210 18274286

[88] Darvesh S, Banfield S, Dufour M, Forrestall KL, Maillet H, Reid GA, Sands D, Pottie IR. A method for the efficient evaluation of substrate-based cholinesterase imaging probes for Alzheimer’s disease. J Enzyme Inhib Med Chem. 2023; 38:2225797. 10.1080/14756366.2023.2225797 38061987 PMC10294744

[89] Rodríguez-Arce E, Saldías M. Antioxidant properties of flavonoid metal complexes and their potential inclusion in the development of novel strategies for the treatment against neurodegenerative diseases. Biomed Pharmacother. 2021; 143:112236. 10.1016/j.biopha.2021.112236 34649360

[90] Maher P. Modulation of the Neuroprotective and Anti-inflammatory Activities of the Flavonol Fisetin by the Transition Metals Iron and Copper. Antioxidants (Basel). 2020; 9:1113. 10.3390/antiox9111113 33187316 PMC7696754

[91] Cheung J, Gary EN, Shiomi K, Rosenberry TL. Structures of human acetylcholinesterase bound to dihydrotanshinone I and territrem B show peripheral site flexibility. ACS Med Chem Lett. 2013; 4:1091–6. 10.1021/ml400304w 24900610 PMC4027152

[92] Brus B, Košak U, Turk S, Pišlar A, Coquelle N, Kos J, Stojan J, Colletier JP, Gobec S. Discovery, biological evaluation, and crystal structure of a novel nanomolar selective butyrylcholinesterase inhibitor. J Med Chem. 2014; 57:8167–79. 10.1021/jm501195e 25226236

[93] Saxena A, Redman AM, Jiang X, Lockridge O, Doctor BP. Differences in active-site gorge dimensions of cholinesterases revealed by binding of inhibitors to human butyrylcholinesterase. Chem Biol Interact. 1999; 119–120:61–9. 10.1016/s0009-2797(99)00014-9 10421439

[94] Kongkaew N, Hengphasatporn K, Shigeta Y, Rungrotmongkol T, Harada R. Preferential Door for Ligand Binding and Unbinding Pathways in Inhibited Human Acetylcholinesterase. J Phys Chem Lett. 2024; 15:5696–704. 10.1021/acs.jpclett.4c00514 38768263

[95] Xu Y, Colletier JP, Weik M, Qin G, Jiang H, Silman I, Sussman JL. Long route or shortcut? A molecular dynamics study of traffic of thiocholine within the active-site gorge of acetylcholinesterase. Biophys J. 2010; 99:4003–11. 10.1016/j.bpj.2010.10.047 21156143 PMC3000518

[96] Wiesner J, Kříž Z, Kuča K, Jun D, Koča J. Influence of the acetylcholinesterase active site protonation on omega loop and active site dynamics. J Biomol Struct Dyn. 2010; 28:393–403. 10.1080/07391102.2010.10507368 20919754

[97] Gilson MK, Straatsma TP, McCammon JA, Ripoll DR, Faerman CH, Axelsen PH, Silman I, Sussman JL. Open “back door” in a molecular dynamics simulation of acetylcholinesterase. Science. 1994; 263:1276–8. 10.1126/science.8122110 8122110

[98] Chinnadurai RK, Saravanaraman P, Boopathy R. Understanding the molecular mechanism of aryl acylamidase activity of acetylcholinesterase - An *in silico* study. Arch Biochem Biophys. 2015; 580:1–13. 10.1016/j.abb.2015.06.002 26072115

[99] Hetényi C, van der Spoel D. Blind docking of drug-sized compounds to proteins with up to a thousand residues. FEBS Lett. 2006; 580:1447–50. 10.1016/j.febslet.2006.01.074 16460734

[100] Bender BJ, Gahbauer S, Luttens A, Lyu J, Webb CM, Stein RM, Fink EA, Balius TE, Carlsson J, Irwin JJ, Shoichet BK. A practical guide to large-scale docking. Nat Protoc. 2021; 16:4799–832. 10.1038/s41596-021-00597-z 34561691 PMC8522653

[101] Maden SF, Sezer S, Acuner SE. Fundamentals of Molecular Docking and Comparative Analysis of Protein–Small-Molecule Docking Approaches. In: Istifli ES, ed. Molecular Docking - Recent Advances. (Rijeka, Croatia: IntechOpen). 2023; 1–24.

[102] Hetényi C, van der Spoel D. Efficient docking of peptides to proteins without prior knowledge of the binding site. Protein Sci. 2002; 11:1729–37. 10.1110/ps.0202302 12070326 PMC2373668

[103] Hetényi C, van der Spoel D. Toward prediction of functional protein pockets using blind docking and pocket search algorithms. Protein Sci. 2011; 20:880–93. 10.1002/pro.618 21413095 PMC3125872

[104] Bálint M, Jeszenői N, Horváth I, van der Spoel D, Hetényi C. Systematic exploration of multiple drug binding sites. J Cheminform. 2017; 9:65. 10.1186/s13321-017-0255-6 29282592 PMC5745209

[105] Waldrop GL. A qualitative approach to enzyme inhibition. Biochem Mol Biol Educ. 2009; 37:11–5. 10.1002/bmb.20243 21567682

[106] Faria OW, de Aguiar MS, de Mello JE, Alvez FL, Luduvico KP, Garcia DN, Schneider A, Masternak MM, Spanevello RM, Stefanello FM. Senolytics prevent age-associated changes in female mice brain. Neurosci Lett. 2024; 826:137730. 10.1016/j.neulet.2024.137730 38485080

[107] Inestrosa NC, Alvarez A, Pérez CA, Moreno RD, Vicente M, Linker C, Casanueva OI, Soto C, Garrido J. Acetylcholinesterase accelerates assembly of amyloid-beta-peptides into Alzheimer’s fibrils: possible role of the peripheral site of the enzyme. Neuron. 1996; 16:881–91. 10.1016/s0896-6273(00)80108-7 8608006

[108] Johnson G, Moore SW. The adhesion function on acetylcholinesterase is located at the peripheral anionic site. Biochem Biophys Res Commun. 1999; 258:758–62. 10.1006/bbrc.1999.0705 10329459

[109] Bartolini M, Bertucci C, Cavrini V, Andrisano V. beta-Amyloid aggregation induced by human acetylcholinesterase: inhibition studies. Biochem Pharmacol. 2003; 65:407–16. 10.1016/s0006-2952(02)01514-9 12527333

[110] Kumar R, Nordberg A, Darreh-Shori T. Amyloid-β peptides act as allosteric modulators of cholinergic signalling through formation of soluble BAβACs. Brain. 2016; 139:174–92. 10.1093/brain/awv318 26525916 PMC4949388

[111] Zueva IV, Vasilieva EA, Gaynanova GA, Moiseenko AV, Burtseva AD, Boyko KM, Zakharova LY, Petrov KA. Can Activation of Acetylcholinesterase by β-Amyloid Peptide Decrease the Effectiveness of Cholinesterase Inhibitors? Int J Mol Sci. 2023; 24:16395. 10.3390/ijms242216395 38003588 PMC10671303

[112] Mukhametgalieva AR, Nemtarev AV, Sykaev VV, Pashirova TN, Masson P. Activation/Inhibition of Cholinesterases by Excess Substrate: Interpretation of the Phenomenological b Factor in Steady-State Rate Equation. Int J Mol Sci. 2023; 24:10472. 10.3390/ijms241310472 37445649 PMC10341919

[113] Fang L, Pan Y, Muzyka JL, Zhan CG. Active site gating and substrate specificity of butyrylcholinesterase and acetylcholinesterase: insights from molecular dynamics simulations. J Phys Chem B. 2011; 115:8797–805. 10.1021/jp112030p 21682268 PMC3135420

[114] Roca C, Requena C, Sebastián-Pérez V, Malhotra S, Radoux C, Pérez C, Martinez A, Antonio Páez J, Blundell TL, Campillo NE. Identification of new allosteric sites and modulators of AChE through computational and experimental tools. J Enzyme Inhib Med Chem. 2018; 33:1034–47. 10.1080/14756366.2018.1476502 29873262 PMC6010107

[115] Carlacci L, Millard CB, Olson MA. Conformational energy landscape of the acyl pocket loop in acetylcholinesterase: a Monte Carlo-generalized Born model study. Biophys Chem. 2004; 111:143–57. 10.1016/j.bpc.2004.05.007 15381312

[116] Hörnberg A, Tunemalm AK, Ekström F. Crystal structures of acetylcholinesterase in complex with organophosphorus compounds suggest that the acyl pocket modulates the aging reaction by precluding the formation of the trigonal bipyramidal transition state. Biochemistry. 2007; 46:4815–25. 10.1021/bi0621361 17402711

[117] Bourne Y, Taylor P, Radić Z, Marchot P. Structural insights into ligand interactions at the acetylcholinesterase peripheral anionic site. EMBO J. 2003; 22:1–12. 10.1093/emboj/cdg005 12505979 PMC140045

[118] Saxena A, Fedorko JM, Vinayaka CR, Medhekar R, Radić Z, Taylor P, Lockridge O, Doctor BP. Aromatic amino-acid residues at the active and peripheral anionic sites control the binding of E2020 (Aricept) to cholinesterases. Eur J Biochem. 2003; 270:4447–58. 10.1046/j.1432-1033.2003.03837.x 14622273

[119] Branduardi D, Gervasio FL, Cavalli A, Recanatini M, Parrinello M. The role of the peripheral anionic site and cation-pi interactions in the ligand penetration of the human AChE gorge. J Am Chem Soc. 2005; 127:9147–55. 10.1021/ja0512780 15969593

[120] 2024 Alzheimer’s disease facts and figures. Alzheimers Dement. 2024; 20:3708–821. 10.1002/alz.13809 38689398 PMC11095490

[121] Barnes LL, Wilson RS, Bienias JL, Schneider JA, Evans DA, Bennett DA. Sex differences in the clinical manifestations of Alzheimer disease pathology. Arch Gen Psychiatry. 2005; 62:685–91. 10.1001/archpsyc.62.6.685 15939846

[122] Sinforiani E, Citterio A, Zucchella C, Bono G, Corbetta S, Merlo P, Mauri M. Impact of gender differences on the outcome of Alzheimer’s disease. Dement Geriatr Cogn Disord. 2010; 30:147–54. 10.1159/000318842 20733307

[123] Reid GA, Darvesh S. Butyrylcholinesterase-knockout reduces brain deposition of fibrillar β-amyloid in an Alzheimer mouse model. Neuroscience. 2015; 298:424–35. 10.1016/j.neuroscience.2015.04.039 25931333

[124] Darvesh S, Reid GA. Reduced fibrillar β-amyloid in subcortical structures in a butyrylcholinesterase-knockout Alzheimer disease mouse model. Chem Biol Interact. 2016; 259:307–12. 10.1016/j.cbi.2016.04.022 27091549

[125] Morley JE, Farr SA, Kumar VB, Armbrecht HJ. The SAMP8 mouse: a model to develop therapeutic interventions for Alzheimer’s disease. Curr Pharm Des. 2012; 18:1123–30. 10.2174/138161212799315795 22288401

[126] Morley JE, Kumar VB, Bernardo AE, Farr SA, Uezu K, Tumosa N, Flood JF. Beta-amyloid precursor polypeptide in SAMP8 mice affects learning and memory. Peptides. 2000; 21:1761–7. 10.1016/s0196-9781(00)00342-9 11150635

[127] Darvesh S, Cash MK, Reid GA, Martin E, Mitnitski A, Geula C. Butyrylcholinesterase is associated with β-amyloid plaques in the transgenic APPSWE/PSEN1dE9 mouse model of Alzheimer disease. J Neuropathol Exp Neurol. 2012; 71:2–14. 10.1097/NEN.0b013e31823cc7a6 22157615 PMC3246090

[128] Koelle GB. The histochemical localization of cholinesterases in the central nervous system of the rat. J Comp Neurol. 1954; 100:211–35. 10.1002/cne.901000108 13130712

[129] Roessmann U, Friede RL. Changes in butyryl cholinesterase activity in reactive glia. Neurology. 1966; 16:123–9. 10.1212/wnl.16.2_part_1.123 5948501

[130] Darvesh S, Leblanc AM, Macdonald IR, Reid GA, Bhan V, Macaulay RJ, Fisk JD. Butyrylcholinesterase activity in multiple sclerosis neuropathology. Chem Biol Interact. 2010; 187:425–31. 10.1016/j.cbi.2010.01.037 20122907

[131] Di Pinto G, Di Bari M, Martin-Alvarez R, Sperduti S, Serrano-Acedo S, Gatta V, Tata AM, Mengod G. Comparative study of the expression of cholinergic system components in the CNS of experimental autoimmune encephalomyelitis mice: Acute vs. remitting phase. Eur J Neurosci. 2018; 48:2165–81. 10.1111/ejn.14125 30144326

[132] Thorne MW, Cash MK, Reid GA, Burley DE, Luke D, Pottie IR, Darvesh S. Imaging Butyrylcholinesterase in Multiple Sclerosis. Mol Imaging Biol. 2021; 23:127–38. 10.1007/s11307-020-01540-6 32926288

[133] Fernández A, Quintana E, Velasco P, Moreno-Jimenez B, de Andrés B, Gaspar ML, Liste I, Vilar M, Mira H, Cano E. Senescent accelerated prone 8 (SAMP8) mice as a model of age dependent neuroinflammation. J Neuroinflammation. 2021; 18:75. 10.1186/s12974-021-02104-3 33736657 PMC7977588

[134] Tha KK, Okuma Y, Miyazaki H, Murayama T, Uehara T, Hatakeyama R, Hayashi Y, Nomura Y. Changes in expressions of proinflammatory cytokines IL-1beta, TNF-alpha and IL-6 in the brain of senescence accelerated mouse (SAM) P8. Brain Res. 2000; 885:25–31. 10.1016/s0006-8993(00)02883-3 11121526

[135] Greig NH, Utsuki T, Ingram DK, Wang Y, Pepeu G, Scali C, Yu QS, Mamczarz J, Holloway HW, Giordano T, Chen D, Furukawa K, Sambamurti K, et al. Selective butyrylcholinesterase inhibition elevates brain acetylcholine, augments learning and lowers Alzheimer beta-amyloid peptide in rodent. Proc Natl Acad Sci USA. 2005; 102:17213–18. 10.1073/pnas.0508575102 16275899 PMC1288010

[136] Lunin SM, Novoselova EG, Glushkova OV, Parfenyuk SB, Novoselova TV, Khrenov MO. Cell Senescence and Central Regulators of Immune Response. Int J Mol Sci. 2022; 23:4109. 10.3390/ijms23084109 35456927 PMC9028919

[137] Ellman GL, Courtney KD, ANDRES V Jr, Feather-Stone RM. A new and rapid colorimetric determination of acetylcholinesterase activity. Biochem Pharmacol. 1961; 7:88–95. 10.1016/0006-2952(61)90145-9 13726518

[138] Darvesh S, Hopkins DA. Differential distribution of butyrylcholinesterase and acetylcholinesterase in the human thalamus. J Comp Neurol. 2003; 463:25–43. 10.1002/cne.10751 12811800

[139] McKhann GM, Knopman DS, Chertkow H, Hyman BT, Jack CR Jr, Kawas CH, Klunk WE, Koroshetz WJ, Manly JJ, Mayeux R, Mohs RC, Morris JC, Rossor MN, et al. The diagnosis of dementia due to Alzheimer’s disease: recommendations from the National Institute on Aging-Alzheimer’s Association workgroups on diagnostic guidelines for Alzheimer’s disease. Alzheimers Dement. 2011; 7:263–9. 10.1016/j.jalz.2011.03.005 21514250 PMC3312024

[140] Hyman BT, Phelps CH, Beach TG, Bigio EH, Cairns NJ, Carrillo MC, Dickson DW, Duyckaerts C, Frosch MP, Masliah E, Mirra SS, Nelson PT, Schneider JA, et al. National Institute on Aging-Alzheimer’s Association guidelines for the neuropathologic assessment of Alzheimer’s disease. Alzheimers Dement. 2012; 8:1–13. 10.1016/j.jalz.2011.10.007 22265587 PMC3266529

[141] Braak H, Alafuzoff I, Arzberger T, Kretzschmar H, Del Tredici K. Staging of Alzheimer disease-associated neurofibrillary pathology using paraffin sections and immunocytochemistry. Acta Neuropathol. 2006; 112:389–404. 10.1007/s00401-006-0127-z 16906426 PMC3906709

[142] Mirra SS, Heyman A, McKeel D, Sumi SM, Crain BJ, Brownlee LM, Vogel FS, Hughes JP, van Belle G, Berg L. The Consortium to Establish a Registry for Alzheimer’s Disease (CERAD). Part II. Standardization of the neuropathologic assessment of Alzheimer’s disease. Neurology. 1991; 41:479–86. 10.1212/wnl.41.4.479 2011243

[143] Karnovsky MJ, Roots L. A “DIRECT-COLORING” THIOCHOLINE METHOD FOR CHOLINESTERASES. J Histochem Cytochem. 1964; 12:219–21. 10.1177/12.3.219 14187330

[144] Forrestall KL, Burley DE, Cash MK, Pottie IR, Darvesh S. Phenothiazines as dual inhibitors of SARS-CoV-2 main protease and COVID-19 inflammation. Can J Chem. 2021; 99:801–11. 10.1139/cjc-2021-0139 PMC7710351 33278462

[145] Forrestall K, Pringle ES, Sands D, Duguay BA, Farewell B, Woldemariam T, Falzarano D, Pottie I, McCormick C, Darvesh S. A phenothiazine urea derivative broadly inhibits coronavirus replication via viral protease inhibition. Antiviral Res. 2023; 220:105758. 10.1016/j.antiviral.2023.105758 38008194

[146] Berman HM, Westbrook J, Feng Z, Gilliland G, Bhat TN, Weissig H, Shindyalov IN, Bourne PE. The Protein Data Bank. Nucleic Acids Res. 2000; 28:235–42. 10.1093/nar/28.1.235 10592235 PMC102472

[147] Forrestall KL, Burley DE, Cash MK, Pottie IR, Darvesh S. 2-Pyridone natural products as inhibitors of SARS-CoV-2 main protease. Chem Biol Interact. 2021; 335:109348. 10.1016/j.cbi.2020.109348 33278462 PMC7710351

