# Supplementary material for: Differential senolytic inhibition of normal versus Aβ-associated cholinesterases: implications in aging and Alzheimer’s disease
Source: Aging (Albany NY). 2025 Mar 29;17(3):822–50. doi: 10.18632/aging.206227 (PMC11984419; doi:10.18632/aging.206227)
Supplement: Supplementary Figure 1 [file aging-17-206227-s001.pdf]

SUPPLEMENTARY FIGURES

Supplementary Figure

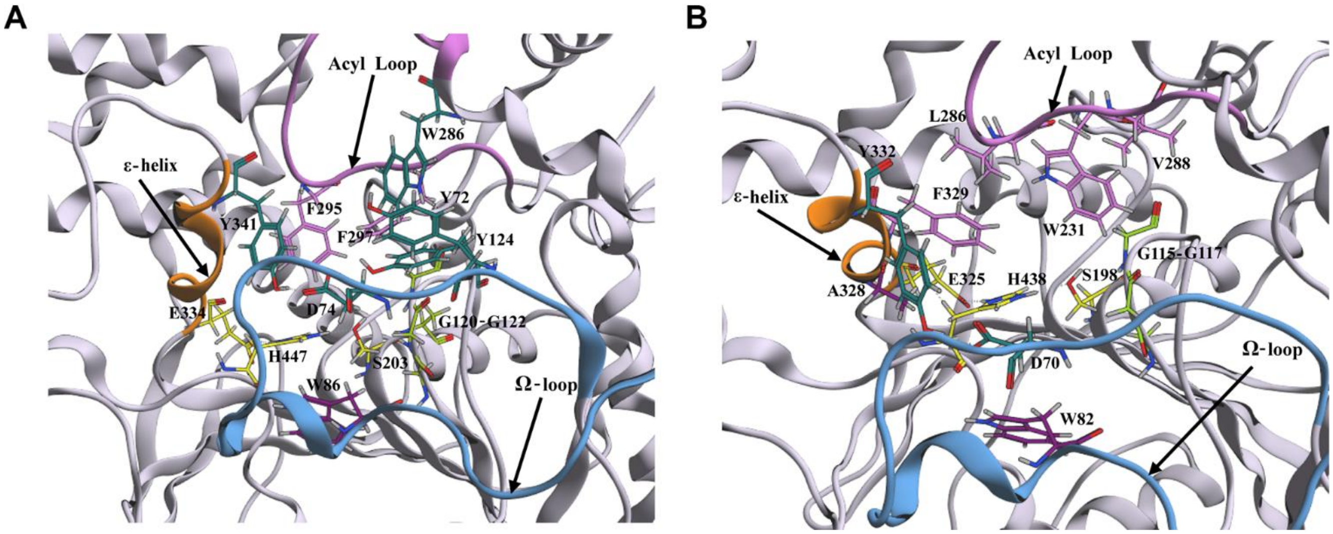

Supplementary Figure 1. Features and key amino acid residues of the active site gorge of acetylcholinesterase (AChE, (**A**) PDD: 4M0E, 2.00 Å) [90] and butyrylcholinesterase (BChE, (**B**) PDB: 4TPK, 2.70 Å). The catalytic triad is shown in yellow ((**A**) S203, E334, H447; (**B**) S198, E325, H438), acyl binding pocket residues are shown in pink ((**A**) F295, F297; (**B**) W231, L286, V288, F329), peripheral anionic site residues are shown in teal ((**A**) Y72, D76, Y124, W286, Y341; (**B**) D70, Y332), π-cationic site residues are shown in purple ((**A**) W86; (**B**) W82, A328), and oxyanion hole residues are shown in lime green ((**A**) G120-G122; (**B**) G115-G117). Acyl loop, Ω-loop, and ε-helix are shown as pink, blue, and orange ribbon structures, respectively. Images were generated with Molecular Operating Environment 2022.02 (Chemical Computing Group ULC, Montreal, Quebec, Canada).
