# Supplementary material for: Differential senolytic inhibition of normal versus Aβ-associated cholinesterases: implications in aging and Alzheimer’s disease
Source: Aging (Albany NY). 2025 Mar 29;17(3):822–50. doi: 10.18632/aging.206227 (PMC11984419; doi:10.18632/aging.206227)
Supplement: Supplementary Tables [file aging-17-206227-s002.pdf]

## SUPPLEMENTARY TABLES

**Supplementary Table 1. Enzyme binding sites identified in blind docking experiments with acetylcholinesterase (AChE; PDB: 4M0E) [90] and butyrylcholinesterase (BChE; PDB: 4TPK) [91] for senolytic and nootropic drugs 1-6 using Molecular Operating Environment 2022.02 (Chemical Computing Group ULC, Montreal, Quebec, Canada).**

| Compound | AChE blind docks | | | | | BChE blind docks | | | | |
|---|---|---|---|---|---|---|---|---|---|---|
| | Site I | Site II | Site III | Site IV | Site V | Site I | Site II | Site III | Site IV | Site V |
| **1** | –[a] | 12/15 | 3/15 | – | – | – | 6/15 | 9/15 | – | – |
| **2** | – | 6/15 | 6/15 | – | 3/15 | – | – | 12/15 | – | 3/15 |
| **3** | 12/15 | 3/15 | – | – | – | 3/15 | – | 9/15 | 3/15 | – |
| **4** | 6/15 | 9/15 | – | – | – | 9/15 | – | 6/15 | – | – |
| **5** | 3/15 | 9/15 | 3/15 | – | – | – | – | 15/15 | – | – |
| **6** | 6/15 | 3/15 | – | 6/15 | – | 6/15 | – | 6/15 | – | 3/15 |

[a]The number of docking poses for 1-6 at each indicated binding site (enzyme active site gorge (I), cholinesterase (ChE) pocket behind acyl loop (II), pocket behind ChE catalytic glutamate (III), pocket behind key ChE active site tryptophan (Back Door, IV), or other site (V)) are shown from triplicate blind docking experiments (3 x 5 poses).

**Supplementary Table 2. *In silico* predicted inhibition constants (*K*i values) for compounds 1-6 with acetylcholinesterase (AChE; PDB: 4M0E, 2.00 Å) [91] and butyrylcholinesterase (BChE; PDB: 4PTK, 2.70 Å) [92].**

| Compound | Predicted AChE $K_i^a$ (µM) | Predicted BChE $K_i^a$ (µM) |
|---|---|---|
| **1** | 27.63* | 0.35* |
| | 126.20† | 3.04† |
| **2** | 16.17* | 1.04† |
| | 61.33† | 5.09⸙ |
| | 110.20⸙ | |
| **3** | 5.32 | 16.49 |
| | 243.12* | 32.46† |
| | | 55.33◊ |
| **4** | 5.14 | 16.66 |
| | 177.24* | 24.76† |
| **5** | 0.73 | 10.30† |
| | 89.63* | |
| | 361.53† | |
| **6** | 11.92 | 16.14 |
| | 191.53* | 42.96† |
| | 299.72◊ | 48.38⸙ |

[a]Sites selected for directed docking were the enzyme active site (Site I, no symbol) or alternate sites (II-V) identified from blind docking experiments (II, *; III, †; IV, ◊; V, ⸙). Molecular docking experiments were completed using Molecular Operating Environment 2022.02 (Chemical Computing Group ULC, Montreal, Quebec, Canada).

**Supplementary Table 3.** ***In silico*** **binding interactions of senolytics 1-5 and nootropic 6 with acetylcholinesterase (AChE, PDB: 4M0E) [91] and butyrylcholinesterse (BChE, PDB: 4TPK) [92] for top site-directed docking poses at cholinesterase (ChE) binding sites I-V.**

| Compound | Enzyme | Binding site (I-V) | Bonding residues[a] | Bond type[a] | Bond length[a] (Å) | Enzyme structure location[b] |
|---|---|---|---|---|---|---|
| 1 | AChE | II | N233 | H-acceptor | 3.08 | Bottom wall of site gorge |
| | | | R296 | H-acceptor | 2.89 | Acyl Loop |
| | | | R296 | H-acceptor | 3.21 | Acyl Loop |
| | | III | K332 | H-acceptor | 3.00 | Site gorge wall |
| | | | R525 | H-acceptor | 3.19 | Site gorge wall |
| | | | R525 | π-H | 4.03 | Site gorge wall |
| | BChE | II | S235 | H-acceptor | 3.03 | Acyl Loop |
| | | | S235 | H-acceptor | 3.04 | Acyl Loop |
| | | | R242 | H-acceptor | 3.48 | Acyl Loop |
| | | | V288 | π-H | 3.95 | Acyl Loop, ABP |
| | | | F357 | H-donor | 3.16 | Site gorge wall |
| | | | N397 | H-acceptor | 3.31 | Site gorge wall |
| | | III | D324 | H-donor | 2.92 | Site gorge wall, beside Ɛ-helix |
| 2 | AChE | II | R296 | H-acceptor | 3.06 | Acyl Loop |
| | | | R296 | H-acceptor | 3.21 | Acyl Loop |
| | | III | R521 | H-acceptor | 3.06 | Site gorge wall |
| | | | R525 | H-acceptor | 3.50 | Site gorge wall |
| | | V | Q181 | π-H | 3.92 | Site gorge wall |
| | BChE | III | K323 | H-acceptor | 3.05 | Site gorge wall |
| | | | V377 | π-H | 4.69 | Site gorge wall |
| | | | D391 | H-donor | 2.99 | Site gorge wall |
| | | | R515 | H-acceptor | 3.11 | Site gorge wall |
| | | V | - | - | - | - |
| 3 | AChE | I | Y72 | H-donor | 2.88 | Ω-loop, PAS |
| | | | W86 | H-donor | 3.09 | Ω-loop, PCS |
| | | | W86 | π-π | 3.77 | Ω-loop, PCS |
| | | | W86 | π-π | 3.79 | Ω-loop, PCS |
| | | | E202 | H-donor | 2.76 | Bottom wall of site gorge |
| | | II | R247 | H-acceptor | 3.02 | Site gorge wall |
| | BChE | I | W82 | π-π | 3.74 | Ω-loop, PCS |
| | | | W82 | π-π | 3.80 | Ω-loop, PCS |
| | | | W82 | H-donor | 2.92 | Ω-loop, PCS |
| | | | E197 | H-donor | 2.78 | Bottom wall of site gorge |
| | | III | Y373 | π-H | 3.99 | Site gorge wall |
| | | | E387 | H-donor | 2.74 | Site gorge wall |
| | | | D391 | H-donor | 2.89 | Site gorge wall |
| | | | R515 | H-acceptor | 3.47 | Site gorge wall |
| | | IV | H77 | π-H | 3.49 | Ω-loop |
| | | | M81 | H-donor | 3.81 | Ω-loop |
| 4 | AChE | I | W86 | π-π | 3.79 | Ω-loop, PCS |
| | | | W86 | π-π | 3.86 | Ω-loop, PCS |
| | | | E202 | H-donor | 2.78 | Bottom wall of site gorge |
| | | II | R296 | π-cation | 3.48 | Acyl Loop |
| | | | R296 | π-cation | 3.94 | Acyl Loop |
| | BChE | I | W82 | π-π | 3.79 | Ω-loop, PCS |
| | | | W82 | π-π | 3.81 | Ω-loop, PCS |

| | | | | | | |
|---|---|---|---|---|---|---|
| | | | E197 | H-donor | 2.77 | Bottom wall of site gorge |
| | | **III** | Y373 | π-H | 3.99 | Site gorge wall |
| | | | E387 | H-donor | 2.74 | Site gorge wall |
| | | | D391 | H-donor | 2.89 | Site gorge wall |
| | | | R515 | H-acceptor | 3.47 | Site gorge wall |
| **5** | AChE | **I** | Y341 | H-π | 4.23 | Ɛ-helix, PAS |
| | | **II** | N233 | H-donor | 3.66 | Bottom wall of site gorge |
| | | **III** | S399 | H-acceptor | 2.96 | Site gorge wall |
| | | | D400 | H-donor | 2.82 | Site gorge wall |
| | | | W442 | H-donor | 2.86 | Opposite S336 of Ɛ-helix |
| | | | R525 | π-cation | 3.75 | Site gorge wall |
| | BChE | **III** | - | - | - | - |
| **6** | AChE | **I** | Y341 | H-π | 3.82 | Ɛ-helix, PAS |
| | | | H447 | π-H | 3.98 | Catalytic Triad |
| | | **II** | R247 | H-acceptor | 3.06 | Site gorge wall |
| | | | R247 | H-acceptor | 3.08 | Site gorge wall |
| | | **IV** | R463 | H-acceptor | 3.36 | Site gorge wall |
| | BChE | **I** | W82 | π-H | 4.36 | Ω-loop, PCS |
| | | | W82 | π-H | 4.43 | Ω-loop, PCS |
| | | **III** | R515 | H-acceptor | 3.33 | Site gorge wall |
| | | | Q517 | H-acceptor | 3.14 | Site gorge wall |
| | | | Q518 | H-acceptor | 3.07 | Site gorge wall |
| | | **V** | Q47 | H-acceptor | 3.13 | Site gorge wall |
| | | | Q176 | H-acceptor | 3.32 | Site gorge wall |

[a]Bonding interactions, type, and lengths were calculated using built-in Molecular Operating Environment (MOE) 2022.02 features (Chemical Computing Group ULC, Montreal, Quebec, Canada).

[b]ChE active regions are also denoted, catalytic active site (CAS), peripheral anionic site (PAS), acyl binding pocket (ABP), π-cationic site (PCS), and oxyanion hole (OAH). Docks that did not show any binding interactions are denoted with dashes.
